# Macrophages fine tune satellite cell fate in dystrophic skeletal muscle of mdx mice

**DOI:** 10.1371/journal.pgen.1008408

**Published:** 2019-10-18

**Authors:** Luca Madaro, Alessio Torcinaro, Marco De Bardi, Federica F. Contino, Mattia Pelizzola, Giuseppe R. Diaferia, Giulia Imeneo, Marina Bouchè, Pier Lorenzo Puri, Francesca De Santa

**Affiliations:** 1 IRCCS Fondazione Santa Lucia (FSL), Rome, Italy; 2 Institute of Biochemistry and Cell Biology (IBBC), National Research Council (CNR), Rome, Italy; 3 Department of Biology and Biotechnology “Charles Darwin”, Sapienza University of Rome, Rome, Italy; 4 Center for Genomic Science of IIT@SEMM, Fondazione Istituto Italiano di Tecnologia (IIT), Milan, Italy; 5 IRCCS European Institute of Oncology (IEO), Milan, Italy; 6 DAHFMO-Unit of Histology and Medical Embryology, Sapienza University of Rome, Rome, Italy; 7 Development, Aging and Regeneration Program, Sanford Burnham Prebys Medical Discovery Institute, La Jolla, California, United States of America; The Jackson Laboratory, UNITED STATES

## Abstract

Satellite cells (SCs) are muscle stem cells that remain quiescent during homeostasis and are activated in response to acute muscle damage or in chronic degenerative conditions such as Duchenne Muscular Dystrophy. The activity of SCs is supported by specialized cells which either reside in the muscle or are recruited in regenerating skeletal muscles, such as for instance macrophages (MΦs). By using a dystrophic mouse model of transient MΦ depletion, we describe a shift in identity of muscle stem cells dependent on the crosstalk between MΦs and SCs. Indeed MΦ depletion determines adipogenic conversion of SCs and exhaustion of the SC pool leading to an exacerbated dystrophic phenotype. The reported data could also provide new insights into therapeutic approaches targeting inflammation in dystrophic muscles.

## Author summary

Muscular dystrophies are a heterogenous group of genetic disorders characterized by muscle wasting, leading to loss of mobility and eventually to death due to respiratory or cardiac failure. Duchenne Muscular Dystrophy (DMD) is one of the most severe dystrophies and is caused by the loss of functional dystrophin protein owing to genetic mutations, consequently, the sarcolemma becomes fragile and susceptible to muscle damage induced by contraction.

Satellite cells (SCs) are skeletal muscle stem cells that mediate the repair process leading to muscle regeneration. Dystrophic muscles undergo continuous cycles of degeneration and regeneration eventually culminating in myofiber loss and deposition of fibrous and fatty connective tissue. Inflammation is always associated with the muscle regeneration process. Among different types of inflammatory cells, mainly macrophages (MΦs) are present in regenerating skeletal muscles and are involved in the regenerative process both after an acute injury and during pathological conditions such as DMD. We focused on the cross-talk between MΦs and SCs in a mouse model of DMD and highlighted a role of MΦs in preserving the SC identity.

## Introduction

Duchenne Muscular Dystrophy (DMD) is one of the most severe dystrophies caused by mutations that lead to the loss of a functional dystrophin protein, a structural protein associated to the muscle fiber membrane. Therefore, dystrophic muscles undergo continuous cycles of degeneration and regeneration that progressively lead to muscle wasting, loss of mobility and eventually to death due to respiratory or cardiac failure [1–3].

Satellite cells (SCs) are bona fide muscle stem cells that are quiescent during homeostasis and are activated in response to acute muscle damage or in chronic degenerative conditions such as DMD [4]. Once activated, SCs are able to both self-renew, in order to maintain the stem cell pool, and to differentiate into myoblasts that fuse with each other and with surrounding fibers to generate new myofibers or repair the damaged ones [5]. The proper expansion and myogenic commitment of SCs is crucial to efficiently counteract muscle degeneration and maintain the SC pool in dystrophic muscles [6,7].

During regeneration of dystrophic muscles, decisions regarding SC fate are regulated by intrinsic mechanisms and extrinsic signals [8]. Dystrophin deficiency in SCs directly affects SC polarity and asymmetric division, thus compromising the self-renewal of myogenic progenitors [9]. Moreover, SCs respond to a large variety of extrinsic signals derived from the regenerative microenvironment that affect muscle stem cell fate. For instance, the induction of Wnt signalling can divert the myogenic cell fate of SCs towards a fibrogenic lineage [10]; SCs undergo mesenchymal-fibrogenic conversion dependent on Tumor Growth Factor-β (TGF-β) signalling [11,12]. The activity of SCs is supported by specialized cell populations resident in skeletal muscles, such as fibroblasts, fibroadipogenic progenitors (FAPs) [13,14] and vascular cells [15], or recruited from the bloodstream in response to muscle damage, represented by immune cells [16].

Inflammation is always associated with the muscle regenerative process in response to muscle degeneration and influences muscle repair. Among different types of inflammatory cells, macrophages (MΦs) are the most abundant immune cells recruited in regenerating skeletal muscles [17,18]. MΦs are highly plastic cells able to respond to the signals derived by the regenerative environment by adopting a broad spectrum of polarization states, not exhaustively characterized by the two extreme states M1 and M2 [19]. In chronic degenerative disease, such as DMD, different MΦ subpopulations coexist at a variable extent depending on the stage of pathology [20]. MΦs not only clear the damaged areas by phagocyting tissue debris, but also sustain the regenerative myogenesis [21]. Indeed, MΦ depletion in acutely injured muscles impairs muscle regeneration [16,22–27], suggesting an active interplay between MΦs and muscle resident cells involved in the repair process.

For instance, several factors, such as Interferon-γ (Ifn-γ) [28], Tumor Necrosis Factor-α (Tnf-α) [29,30], Interleukin-6 (IL-6) [31], Insulin growth factor-1 (Igf-1) [32], Granulocyte-colony stimulating factor (G-csf) [33], Klotho (Klb) [34,35] and Adamts1 [36] positively affect SC proliferation, while others, such as Growth Differentiation Factor 3 (Gdf3) [37] or Tgf-β [38,39] sustain the myogenic differentiation of SCs. However, the functional significance of the SCs-MΦs cross-talk is only partially elucidated. Anti-inflammatory drugs are a promising therapeutic approach to attenuate symptoms of muscular dystrophies, including DMD. Indeed, corticosteroids (prednisolone or deflazacort), that have an anti-inflammatory activity, are currently the standard care for treatment of DMD and other muscular dystrophies; however, the side effects of these drugs often outweigh their beneficial effects [40–44]. Therefore, the importance of tackling the interplay between SCs and MΦs in chronic muscle disease like DMD also lies in the need to finely assess the potential or the effectiveness of inflammation-based therapeutic approaches for treating DMD.

With our data we describe in dystrophic skeletal muscle a shift in identity of muscle stem cells dependent on the crosstalk between MΦs and SCs. Indeed, the characterization of a dystrophic mouse model of transient MΦ depletion, named mdx^ITGAM-DTR^ revealed a critical role of MΦs in preserving SC identity and preventing precocious exhaustion of SCs, sustaining, in turn, proper muscle regeneration. Specifically, MΦ depletion exacerbates the dystrophic phenotype, impairs the proliferation/differentiation balance of myogenic progenitors and causes adipogenic conversion of SCs. These findings could also provide new insights into therapeutic approaches targeting inflammation in dystrophic muscles.

## Results

### Macrophage depletion compromises satellite cell potential and muscle regeneration in dystrophic mice

We investigated the role of macrophages (MΦs) in regeneration of dystrophic muscles by taking advantage of a transgenic mouse model that allows to transiently deplete macrophages (B6.FVB-Tg ITGAM-DTR/EGFP mice; The Jackson Laboratory) [45], referred to as ITGAM-DTR. In ITGAM-DTR mice, monocyte/ MΦ population can be depleted by Diphtheria Toxin (DT) injection. Among the immune cells also granulocytes, mainly neutrophils, express ITGAM/CD11b, but are not targeted by DT in ITGAM-DTR mice [16,45,46], which thus represent a tool to specifically study the role of monocytes/ MΦs in different processes. To tackle the role of MΦs in regeneration of dystrophic muscles, ITGAM-DTR mice were crossed with mdx mice, a widely used mouse model of Duchenne Muscular Dystrophy (DMD), following the breeding scheme reported in S1A Fig. mdx^ITGAM-DTR^ mice are viable, fertile, normal in size and, in the absence of DT injection, show a dystrophic phenotype overlapping that of mdx mice.

Either DT or PBS vehicle was administered to mdx^ITGAM-DTR^ mice by intramuscular (im) injection in the Tibialis Anterior (TA) and the Gastrocnemius (GA). To gain substantial and prolonged MΦ depletion in muscles of mdx^ITGAM-DTR^ mice we adopted the 15-day long DT injection schedule reported in S1B Fig. Then we used a fluorescence activated cell sorting (FACS) approach to isolate both macrophages and muscle-resident cells, namely satellite cells (SCs) and fibroadipogenic progenitors (FAPs), from hind limb muscles of PBS or DT-injected mdx^ITGAM-DTR^ mice; the gating strategy is reported in S1C Fig.

15 days after the first DT injection, MΦs were sorted as Lineage^+^(CD31^+^, CD45^+^, Ter119^+^) (Lin^+^)/CD11b^+^/F4/80^+^ cells in order to verify successful MΦ depletion in dystrophic muscles, highly enriched in infiltrating MΦs (S1D and S1E Fig).

Macrophage depletion was also evaluated by FACS at intermediate time points, specifically d3, d7 and d11, these correspond to the day prior the scheduled DT injection, in order to check that MΦ depletion was maintained during the 15 days of treatment (S1F Fig).

The robust MΦ depletion at d15 was also confirmed by immunostaining of TA cryosections with an antibody directed against the MΦ-specific marker F4/80 (S1G Fig).

We also verified that neutrophils were not depleted by analyzing Ly6G (GR1) positive cells by FACS (S1H Fig). Furthermore, to verify whether DT *per se* did not affect MΦs, DT was also injected in mdx mice where, indeed, the relative percentage of MΦs was not altered (S1J and S1K Fig).

**Fig 1:**
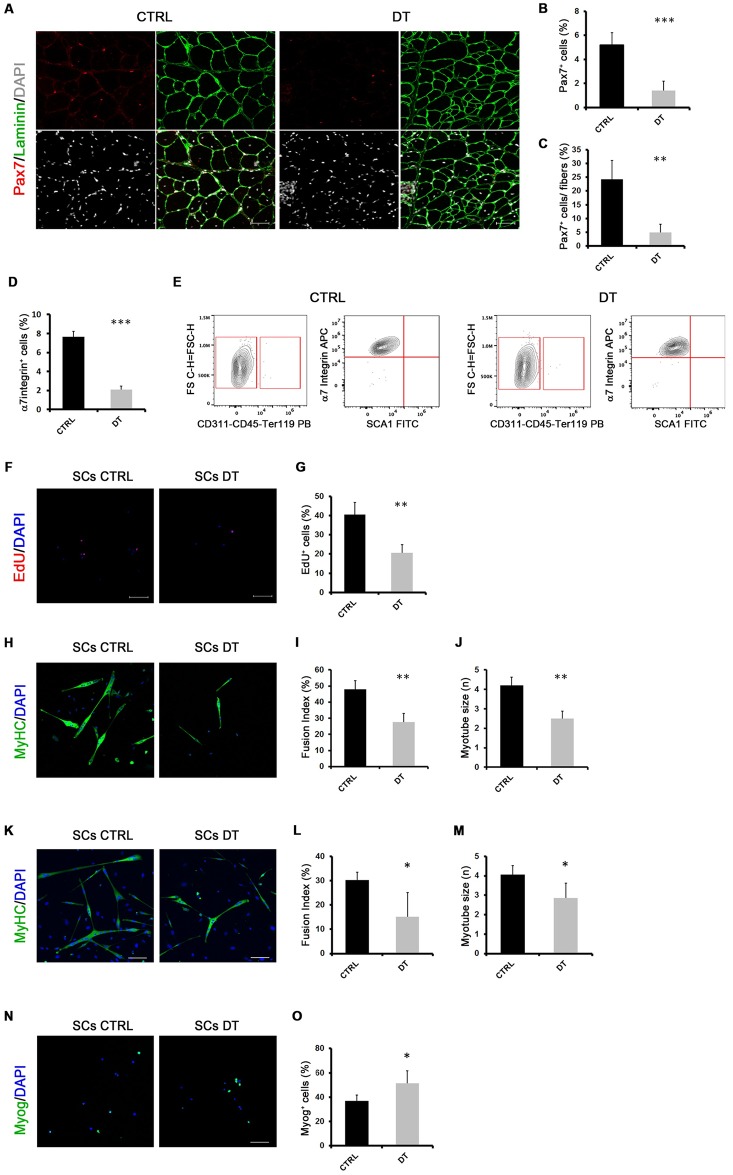
MΦ depletion compromises satellite cell potential in dystrophic mice. (**A, B, C**) Representative images of double staining anti-laminin (green) and anti-Pax7 (red) of TA cryosections of mdx^ITGAM-DTR^ mice injected with PBS (CTRL) or DT. Nuclei were counterstained with DAPI (white). In the graphs are reported the percentage of Pax7^+^ cells relative to total cells or the percentage of Pax7^+^ cells relative to fibers; values are mean ± SEM; n = 3 animals for each experimental group; unpaired t test was used for comparison (**, P<0.01; ***, P<0.001). Scale bar = 50 μm. (**D**) Relative percentage of SCs sorted from muscles of mdx^ITGAM-DTR^ mice injected with PBS or DT. Cells isolated from hind limb muscles were first separated into hematopoietic lineage positive (Lin^+^) and hematopoietic lineage negative (Lin^-^) (Lin: CD45, CD31 and Ter119) cells. SCs were then sorted as α7Integrin^+^ (α7^+^)/Sca1^-^/ Lin^-^; the percentage of cells is reported as relative to whole mononucleated cells; values are mean ± SEM (n = 3 biological replicates for each experimental group; each replicate was the pool of 2 mice); unpaired t test was used for comparison (***, P<0.001). (**E**) Purity check of SCs sorted from muscles of mdx^ITGAM-DTR^ mice injected with PBS (CTRL) or DT. Freshly sorted SCs were analyzed by flow cytometry (CytoFLEX, Beckman Coulter) and showed purity ≥98%. (**F, G**) Sorted SCs were cultured in growth medium (GM) for 2 days and then EdU were added for 4 h in fresh GM before the fixation. EdU-incorporating SCs were then stained using Click-iT EdU Alexa Fluor Imaging Kit and 4′,6-diamidino-2 phenylindole (DAPI). Proliferating cells were counted checking the co-localization of DAPI and EdU positivity. In the graph is reported the quantification: values are mean ± SEM (n = 3 biological replicates for each experimental group; each replicate was the pool of 2 mice); unpaired t test was used for comparison (**, P<0.01). (**H, I, J**) Representative images of MyHC staining of SCs isolated from mdx^ITGAM-DTR^ mice injected with PBS (SCs CTRL) or DT (SCs DT) and differentiated *ex vivo*. The cells were cultured in growth medium for 48 hours and then shifted in differentiation medium for further 48h. Nuclei were counterstained with DAPI (blue). In the graphs are reported the quantification of two skeletal muscle differentiation parameters: fusion index (percentage of nuclei within myotubes: myotube = nuclei ≥2) and myotube size (mean of number of nuclei into myotubes). Data are represented as mean ± SEM (n = 4 independent experiments); unpaired t test was used for comparison (**, P<0.01). Scale bar = 100 μm. (**K, L, M**) Representative images of MyHC staining of SCs isolated from mdx^ITGAM-DTR^ mice injected with PBS (SCs CTRL) or DT (SCs DT) and high-density differentiated *ex-vivo*. The cells were cultured in growth medium for 48 hours and then seeded at high density (60000 cells/cm2) and, after few hours, once adherent, shifted in differentiation medium for a further 48h. Nuclei were counterstained with DAPI (blue). In the graphs are reported the quantification of fusion index (percentage of nuclei within myotubes: myotube = nuclei ≥2) and myotube size (mean of number of nuclei into myotubes). Data are represented as mean ± SEM (n = 4 independent experiments); unpaired t test was used for comparison (*, P<0.05). Scale bar = 100 μm. **(N, O)** Representative images of Myogenin (Myog) staining of SCs isolated from mdx^ITGAM-DTR^ mice injected with PBS (SCs CTRL) or DT (SCs DT) and seeded *ex vivo* in growth medium for 4 days. Nuclei were counterstained with DAPI (blue). In the graph is reported the quantification of Myog^+^ cells. Data are represented as mean ± SEM (n = 4 independent experiments); unpaired t test was used for comparison (*, P<0.05). Scale bar = 100 μm.

Next, we analyzed the effect of a 15-day MΦ depletion on muscle resident satellite cells (SCs). The analysis of Pax7^+^ cells on muscle cryosections confirmed a drastic reduction of SCs in mdx^ITGAM-DTR^ mice upon MΦ depletion (Fig 1A, 1B and 1C). Pax7 is a marker of adult SCs, specifically expressed in proliferating and self-renewing satellite cells, whose inactivation leads to a severe impairment in regeneration and loss of satellite cells [47,48].

We also isolated by FACS SCs from PBS or DT-injected hindlimb muscles of 12 week-old mdx^ITGAM-DTR^ mice, identified as α7Integrin^+^ (α7^+^)/ Stem cell antigen^-^ (Sca1)^-^/CD31^-^, CD45^-^, Ter119^-^ (Lin^-^) cells and we found that the relative number of SCs was strongly reduced upon MΦ depletion (Fig 1D). Moreover, we verified the purity of SCs by cytofluorimetric analysis of freshly sorted cells in order to assess the level of contaminating cells in SCs DT sample, in which the impact of contaminating cells could be higher, considering the significant decrease in SC number upon DT injection. The purity check confirmed a similar and high percentage of purity (about 98%) in SCs CTRL and SCs DT (Fig 1E). Therefore, the decrease in Pax7^+^ cells suggested that MΦ depletion could compromise SC expansion and self-renewal leading to exhaustion of the SC pool.

SCs isolated from mdx^ITGAM-DTR^ mice were cultured *ex vivo*. The EdU (5–ethynyl–2′–deoxyuridine) incorporation revealed a reduced proliferative rate of SCs derived from DT-injected mdx^ITGAM-DTR^ mice (SCs DT) compared to control SCs (SCs CTRL) (Fig 1F and 1G), which could reflect either a proliferation deficit or precocious myogenic differentiation. Moreover, upon induction of differentiation SCs derived from a MΦ-depleted muscle niche (SCs DT) showed evident differentiation defects compared to control SCs, both in terms of fusion index and myotube size (Fig 1H, 1I and 1J).

Taking into consideration the observed proliferative defect, the differentiation level could also be affected by the reduced cell density of SCs DT. Therefore, we performed a differentiation assay of cells seeded at high-density, in order to evaluate the differentiation efficiency independently of cell number at the moment of the shift in differentiation medium. We found that SCs DT showed differentiation defects also in high-density culturing condition, indicating an intrinsically impaired differentiation potential (Fig 1K, 1L and 1M).

Moreover, we also evaluated early stages of myogenic differentiation prior to fusion by analyzing the expression of Myogenin (Myog) and we verified that, despite lower density of SCs DT, the percentage of Myog^+^ cells was higher in SCs DT compared to SCs CTRL, suggesting a precocious onset of differentiation (Fig 1N and 1O).

Next, we analyzed the effect of MΦ depletion on the dystrophic muscle phenotype of 12 week-old mdx^ITGAM-DTR^ mice, in terms of regeneration, fibrosis and fat deposition. In DT-treated mdx^ITGAM-DTR^ mice the histological analysis showed a disordered muscle architecture with evident interstitial spaces and infiltrating cells among fibers (S2A Fig). Furthermore, the measurement of myofiber cross sectional area (CSA) demonstrated a decrease in the mean fiber area (S2B Fig), due to a significant increase in the number of smaller fibers and a decrease of intermediate and large fibers in dystrophic muscles depleted of MΦs (S2C Fig).

We then assayed the expression of embryonic Myosin Heavy Chain (eMyHC), an early myosin isoform transiently expressed by newly formed regenerating fibers (S2D Fig). The increased number of eMyHC-positive fibers revealed a strong increase of newly generated fibers in DT-injected muscles of mdx^ITGAM-DTR^ suggesting an enhanced activation of the regenerative process upon MΦ depletion (S2E Fig).

Specifically, the total number of myofibers showed a slight, though not significant, increase in DT-injected mice (S2F Fig), presumably due to the high number of regenerating fibers; however, the quantification of myonuclei showed a reduction in the mean number of myonuclei contained in eMyHC^+^ fibers of DT-treated mdx^ITGAM-DTR^ mice compared to control mice (S2G Fig). The above observations suggest that a reduced supply of myonuclei or lack of fusion could compromise the maturation of myofibers resulting in a decreased size of myofibers upon MΦ depletion. Interestingly, the reduced number of myonuclei in regenerating fibers of DT-injected mdx^ITGAM-DTR^ mice mirrors the fusion defect of SCs DT observed *in vitro*.

The expression analysis by qRT-PCR of whole TA muscles from PBS or DT-injected mdx^ITGAM-DTR^ mice confirmed the induction of myogenic regulatory factors and early myosin isoforms, such as Myogenin, Mrf4 and Myh3 (eMyHC), in MΦ-depleted dystrophic muscles (S2H Fig), but several late muscle markers crucial for myofibril assembly and contraction, such as adult myosin isoforms (Myh1, MyH4), muscle creatine kinase (Mck) and members of myozenin and tropomodulin families (Myoz3, Tmod1) were strongly reduced in DT-injected mdx^ITGAM-DTR^ mice (S2I Fig), suggesting an impairment in myofiber maturation.

Sirius Red staining revealed a significantly increased deposition of fibrotic tissue in mdx^ITGAM-DTR^ mice upon DT injection, suggesting an important role of MΦs in fibrotic degeneration of muscles at the regenerative stage of dystrophy (S3A and S3B Fig). Moreover, we analyzed adipose tissue deposition on muscle cryosections of PBS or DT-injected mdx^ITGAM-DTR^ mice. Specifically, TA muscles were stained with either Oil Red O dye (S3C and S3D Fig), specific for triglycerides, or with antibodies specific for Perilipin, a late marker of adipogenic differentiation (S3E Fig) which revealed evident signals of fat deposition in dystrophic muscles from mdx^ITGAM-DTR^ mice upon MΦ depletion. The fibro-adipogenic degeneration was further confirmed at the molecular level through expression analysis of fibrotic and adipogenic differentiation markers in whole TA muscles derived from mdx^ITGAM-DTR^ mice (S3F Fig). The increase in ECM and fat deposition indicates a less efficient compensatory regeneration presumably leading to a compromised functionality of muscles upon MΦ depletion.

To verify that DT *per se* did not affect dystrophic muscles, DT was also injected in mdx mice where, indeed, the muscle architecture was not altered (S4A and S4B Fig) and fibrotic tissue deposition of mdx mice was not increased by DT injection (S4C and S4D Fig).

Collectively, these data indicate that MΦ depletion triggers the muscle regenerative program in dystrophic muscle, evidenced by an increase in the percentage of eMyHC^+^ fibers (S2D and S2E Fig) and induction of early muscle differentiation markers (S2H Fig); however, the histopathological analysis of hind limb muscle demonstrates an exacerbated dystrophic phenotype characterized by a decrease of the mean myofiber size and an increase of fibrosis and fat deposition. Moreover, the reduced number of myonuclei contained in eMyHC^+^ fibers and the strong reduction of muscle differentiation markers associated with the assembly and activity of myofibrils suggest an altered timing of the myogenic process and a less efficient maturation of fibers. This phenotype is also associated with a reduced number of SCs *in vivo* and both proliferation and differentiation defects of SCs *ex vivo*.

### Transcriptome analysis reveals adipogenic conversion of SCs upon MΦ depletion in dystrophic muscles

To investigate the molecular pathways underlying the evident effects of MΦ depletion on muscle progenitor cells, we performed genome-wide expression analysis of freshly sorted SCs derived from mdx^ITGAM-DTR^ mice injected with PBS (SCs CTRL) or DT (SCs DT) following the experimental scheme reported in S1B Fig.

**Fig 2:**
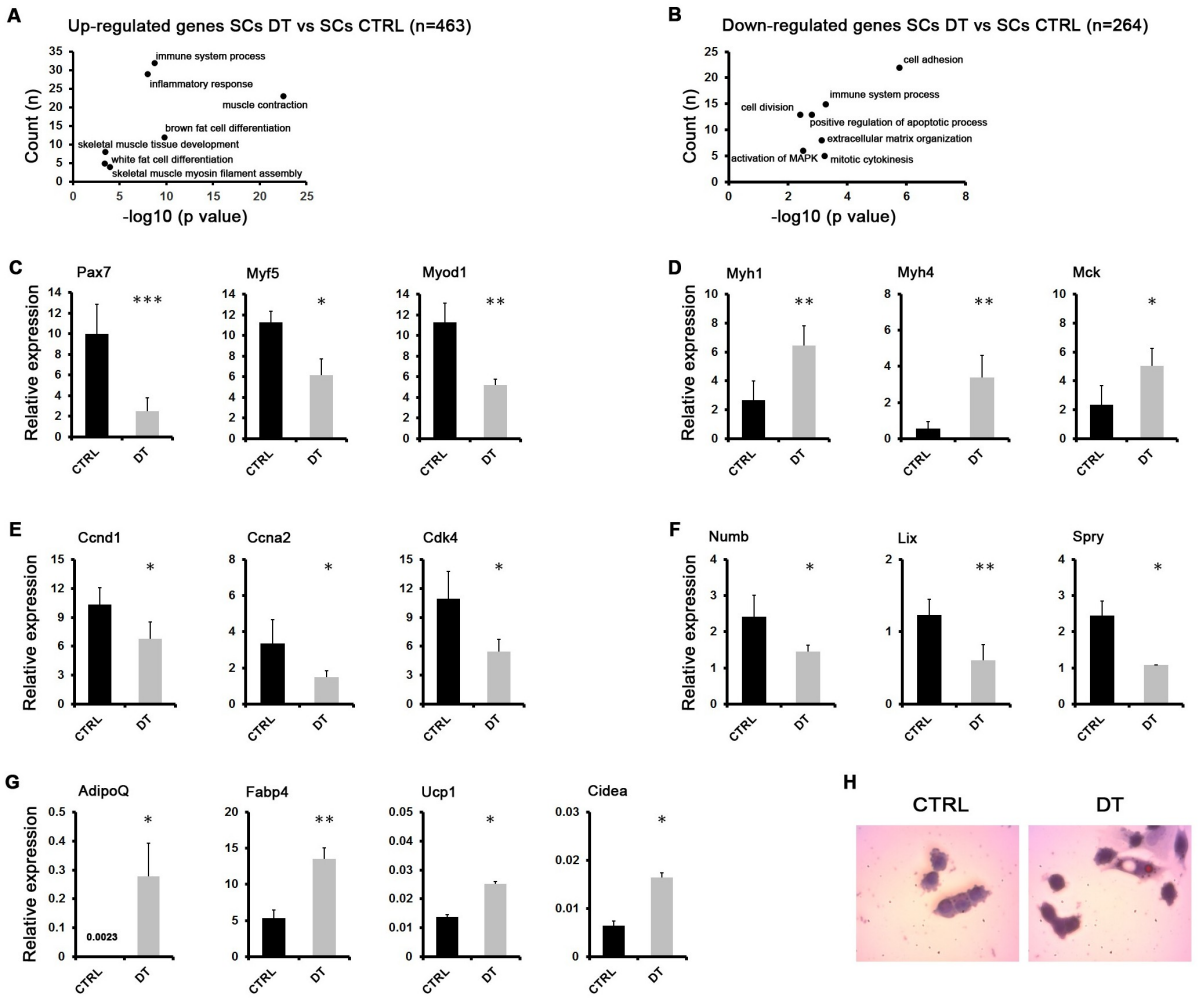
*Transcriptome analysis reveals adipogenic conversion of SCs upon* MΦ *depletion in dystrophic muscles*. (**A, B**) Total RNA from SCs derived from PBS-injected (SCs CTRL) or DT- injected (SCs DT) mdx^ITGAM-DTR^ mice was used for RNA-Seq (n = 2 biological replicates for each experimental group; each replicate is the pool of 2 mice). Selected representative GO biological processes in upregulated (A) and downregulated genes (B) in SCs derived from DT-injected mdx^ITGAM-DTR^ mice compared to CTRL mice, identified by DAVID 6.8 are shown. The graph displays for each GO term the obtained p value (expressed as −log10) on the x axis and the number of genes included (count), on the y axis. (**C, D, E, F, G**) Expression analysis by qRT-PCR of myogenic (C, D), proliferation (E), self-renewal (F) and adipogenic (G) markers in SCs isolated from mdx^ITGAM-DTR^ mice injected with PBS (CTRL) or DT. The data are reported as relative to housekeeping gene TBP, and represented as mean ±SEM (n = 3 or 4 biological replicates for each experimental group; each replicate was the pool of 2 mice); unpaired t test was used for comparison (*, P<0.05; **, P<0.01; ***, P<0.001). (**H**) Representative images of *in vitro* cultures of SCs CTRL and SCs DT cells isolated from mdx^ITGAM-DTR^ mice injected with PBS (CTRL) or DT. The cells were cultured in SCs growth medium for 24 hours and then stained by Oil Red O dye and counterstained with Hematoxilin.

Pairwise analysis of the transcriptomes of SCs CTRL and SCs DT revealed 727 differentially expressed genes (DEGs), either upregulated (n = 463) or downregulated (n = 264) in SCs DT versus SCs CTRL, which were evaluated for enrichment of Gene Ontology (GO) terms (Fig 2A and 2B). The most representative biological processes included the immune response, muscle and adipogenic differentiation for the upregulated genes (Fig 2A) and proliferation and adhesion-related processes for the downregulated genes (Fig 2B).

Validation of RNA-Seq data by RT-qPCR confirmed a strong modulation of muscle-specific genes in SCs upon MΦ depletion. Specifically, on one hand we found a robust decrease of markers crucial for activation and myogenic commitment of SCs, such as Pax7, Myf5 and MyoD, (Fig 2C) and, on the other hand, we observed a significant increase of late markers of skeletal muscle differentiation, such as Mrf4 (Myf6), Mck and different isoforms of adult MyHC genes (Myh1, Myh4) (Fig 2D), supporting the hypothesis of precocious differentiation of SCs upon MΦ depletion [49].

Moreover, SCs derived from MΦ-depleted dystrophic muscles of mdx^ITGAM-DTR^ mice showed downregulation of genes involved in cell cycle progression, such as Cyclin D1 (Ccnd1), Cyclin A (Ccna2) and ckd4 (Fig 2E), or in quiescence of adult SCs, such as Numb, Spry and Lix1 (Fig 2F); this expression pattern was consistent with the reduced cell density observed in *ex vivo* cell cultures (Fig 1F and 1G). Collectively, these data suggest an impaired self-renewal capacity and precocious differentiation of SCs upon MΦ depletion leading to exhaustion of the SC pool and exacerbation of dystrophic phenotype. These effects are reminiscent of what was previously observed in Pax7-null SCs [47,49].

Previous reports suggested that SCs might adopt an alternative adipogenic fate. In particular, *in vivo* lineage tracing experiments demonstrated that skeletal muscle and brown fat cells, but not white fat cells, derive from Myf5^+^ precursor cells and this switch of cell fate is controlled by the transcriptional regulator PRDM16 [50,51].

In this context, the top-ranking GO categories for upregulated genes in SCs DT included fat cell differentiation-related terms. In fact, in SCs DT mice we could appreciate an increased expression of adipogenic markers (Fig 2G), in concomitance with MyoD inhibition (Fig 2C), Importantly, MyoD inhibition is a crucial event in promoting lineage switching of SCs toward a brown adipogenic phenotype [49,52,53], and, in this context, Ucp1 and Cidea (Fig 2G) represent brown fat-specific markers [54].

We also analyzed the adipogenic conversion of SCs DT at single cell level by Oil Red O staining of *ex vivo* cultured SCs DT and we found several cells containing Oil Red positive dots, undetectable in SCs CTRL cells (Fig 2H).

Collectively, these data suggest that MΦ depletion on the one hand compromises the self-renewal of SCs leading to precocious differentiation and, on the other hand, triggers in SCs a mesenchymal alternative program determining the adoption of a brown adipogenic fate.

### A novel cell population revealed in dystrophic muscles upon MΦ depletion

**Fig 3:**
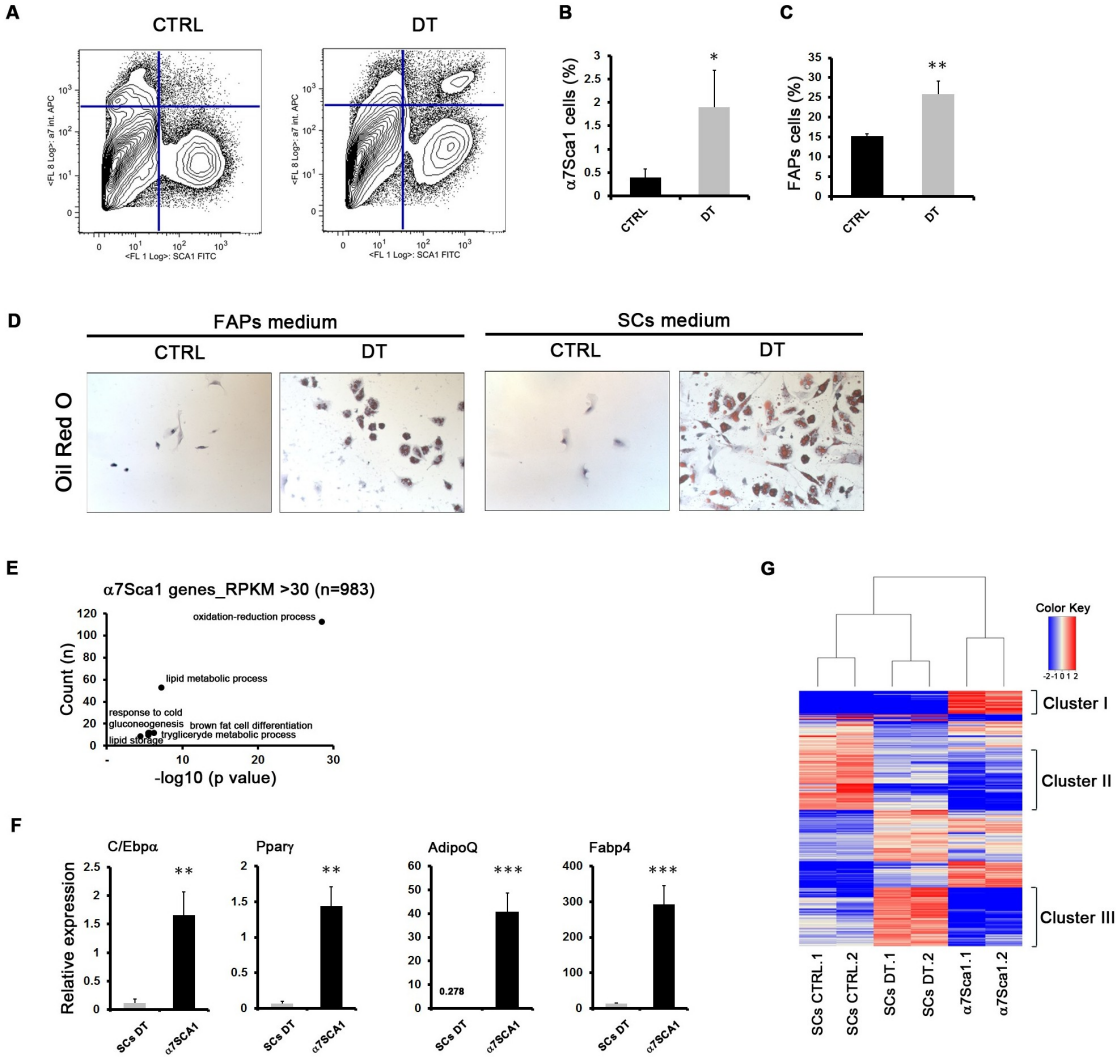
*A novel cell population revealed in dystrophic muscles upon* MΦ *depletion*. (**A**) FACS plot representation of cell sorted from muscles of mdx^ITGAM-DTR^ mice. Cells were first separated into hematopoietic lineage positive (Lin^+^) and negative (Lin^-^) (Lin: CD45, CD31 and Ter119) cells. SCs and FAPs were sorted as described in S1C Fig. A double positive cell population α7^+^/ Sca1^+^/Lin^-^ (α7Sca1 cells), was sorted from DT-injected mdx^ITGAM-DTR^ mice and was not significantly detectable in PBS-injected mdx^ITGAM-DTR^ mice. (**B-C**) Graphs showing the percentage of α7Sca1 cells (B) and FAPs (C) sorted from mdx^ITGAM-DTR^ mice injected with PBS (CTRL) or DT. The percentages are reported as relative to whole mononucleated cells. Values are mean ± SEM (n = 3 or 4 biological replicates for each experimental group; each replicate was the pool of 2 mice); unpaired t test was used for comparison (*, P<0.05; **, P<0.01). (**D**) Representative images of in vitro culture of α7Sca1 cells isolated from mdx^ITGAM-DTR^ mice injected with PBS (CTRL) or DT. The cells were cultured both in FAPs growth medium and in SCs growth medium for 36 hours and then cells were stained by Oil Red O dye and counterstained with Hematoxilin. Scale bar = 50 μm (**E**) Total RNA from α7Sca1 cells derived from DT- injected mdx^ITGAM-DTR^ mice was used for RNA-Seq (n = 2 biological replicates for each experimental group; each replicate was the pool of 2 mice). Selected representative GO biological processes in highly expressed genes (RPKM≥30) of α7Sca1 cells identified by DAVID 6.8 are shown. The graph displays for each GO term the obtained p value (expressed as −log10) on the x axis and the number of genes included (count), on the y axis. (**F**) Expression analysis by qRT-PCR of adipogenic markers in SCs and α7Sca1 cells isolated from DT-injected mdx^ITGAM-DTR^ mice. The data are reported as relative to housekeeping gene TBP, and represented as mean ±SEM (n = 3 biological replicates for each experimental group; each replicate was the pool of 2 mice); unpaired t test was used for comparison (**, P<0.01; ***, P<0.001). (**G**) Heat map of 727 deregulated genes in SCs (DEGs DT vs CTRL) isolated from mdx^ITGAM-DTR^ mice identified by RNA-Seq, compared to the expression pattern of α7Sca1 cells. The heat map shows the differential expression of DEGs in SCs and α7Sca1 cells as relative to the mean expression value of all six samples.

Interestingly, FACS isolation revealed that MΦ depletion was also associated with the appearance of a cell population, referred to as α7Sca1 cells, that co-expresses both α7integrin and Sca1 (Stem cell antigen) surface markers, usually used as specific and mutually exclusive markers to sort SCs or fibroadipogenic progenitors (FAPs) [13,14], respectively (Fig 3A and 3B). In PBS-injected mdx^ITGAM-DTR^ mice, α7Sca1 cells were not significantly detectable (Fig 3A). In the FACS plot is also evident the already reported reduction of SCs upon MΦ depletion (Fig 1D). Moreover, we could also appreciate an increase in the FAPs population (Fig 3C), sorted as Sca1^+^/α7Integrin^-^(α7^-^)/(Lin^-^) cells, suggesting that the observed increase of fibrosis and fat deposition (S3 Fig) may reflect an enhanced *in vivo* amplification of FAPs, considered the main source of matrix-producing fibroblasts [13,14]. In mdx mice α7Sca1 cells were not detected (S5A Fig) and the abundance of SCs and FAPs was not affected by DT injection (S5B and S5C Fig), ruling out an effect of DT injection, independent from MΦ depletion.

We analysed α7Sca1 cells *ex vivo*, by seeding them in SC or FAP growing medium. These cells, which were significantly detectable only in MΦ-depleted mdx^ITGAM-DTR^ mice, failed to proliferate *ex vivo*, showed an enlarged adipocyte-like morphology even in the absence of any adipogenic stimulus, and displayed several droplets of triglycerides when stained with Oil Red O dye (Fig 3D). The transcriptome of α7Sca1 cells confirmed the adipocyte-like phenotype: in fact, representative GO categories for genes having an RPKM over 30 included terms related to adipocyte differentiation and adipose tissue function (Fig 3E). The validation of transcriptome data by qRT-PCR confirmed the adipocyte-like phenotype of α7Sca1 cells showing a consistent expression of adipogenic markers compared to SCs DT isolated from DT-injected mdx^ITGAM-DTR^ mice (Fig 3F).

Comparative analysis of the deregulated genes in SCs and α7Sca1 transcriptome (Fig 3G) identified few clusters of genes belonging to a restricted number of biological processes, as evaluated by GO analysis (S5D, S5E and S5F Fig). Specifically, cluster I mainly included adipogenic markers (S5D Fig), such as AdipoQ, Fabp4, Adig, which were highly expressed in α7Sca1 cells. These markers were also induced in SCs DT compared to SCs CTRL (Fig 2G), although hidden in the heat map by the impressive expression in α7Sca1 cells.

Moreover, a second cluster (cluster II) was represented by genes downregulated in SCs DT and not expressed in α7Sca1 cells, mainly involved in cell proliferation, cell adhesion and regulation of cell differentiation (S5E Fig, Fig 2A, 2E and 2F). On the contrary, the last group (cluster III) included genes strongly upregulated in SCs DT and repressed in α7Sca1 cells; these genes are essentially involved in muscle development and differentiation (S5F Fig and Fig 2D).

Transcriptome analysis and comparison suggest that SCs DT show the expression of non muscle-related genes, mainly adipogenic markers, and the repression, among others, of proliferative genes, an expression profile in common with α7Sca1 cells; instead SCs DT share the expression of muscle-related genes with SCs CTRL, in particular, showing an upregulation of the late ones.

### α7Sca1 cells derive from SCs upon MΦ depletion in dystrophic muscles

**Fig 4:**
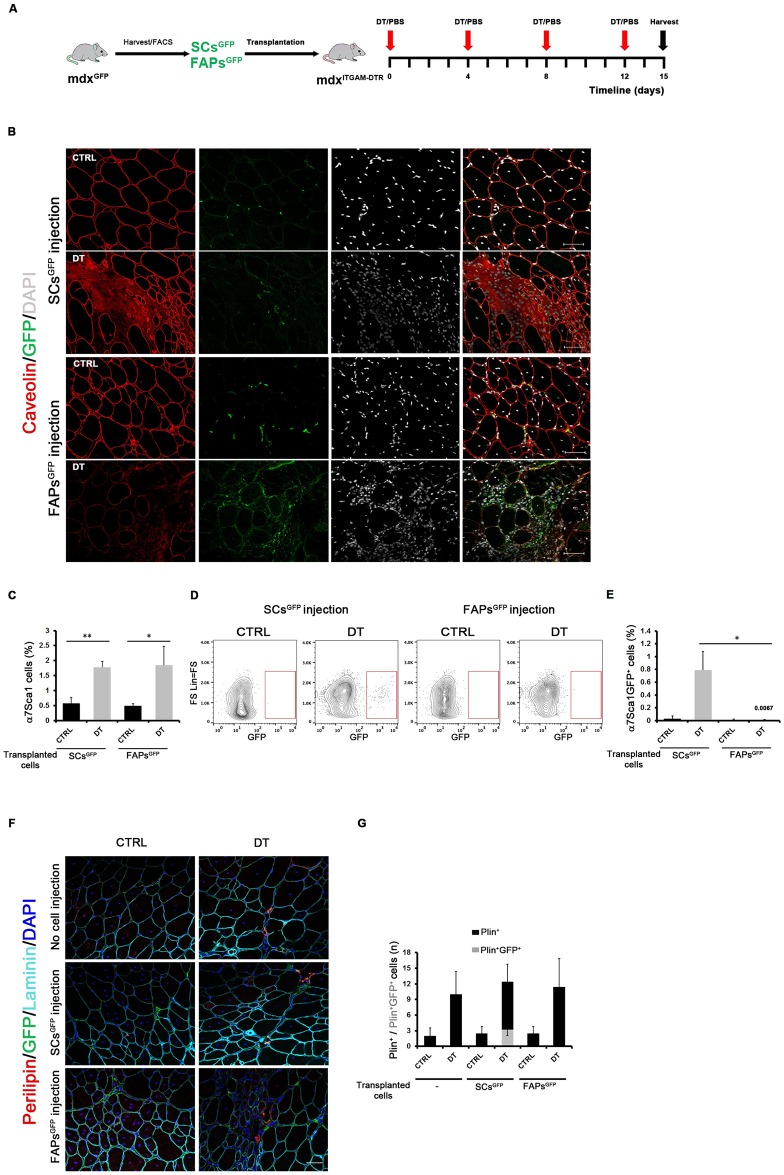
α7Sca1 cells derive from SCs upon MΦ depletion in dystrophic muscles. (**A**) Schematic representation of transplantation experiment. A detailed description is reported in the Results section. (**B**) Representative images of double staining anti-caveolin (red) and anti-GFP (green) of TA cryosections of mdx^ITGAM-DTR^ mice transplanted with SCs^GFP^ and FAPs^GFP^ and injected with PBS (CTRL) or DT. Nuclei were counterstained with DAPI (white); n = 3 animals for each experimental group. Scale bar = 50 μm. (**C**) Percentage of α7Sca1 cells purified from Lin^-^ cell population of PBS or DT-injected mdx^ITGAM-DTR^ mice transplanted with SCs^GFP^ and FAPs^GFP^; the percentage of α7Sca1 cells is reported as relative to whole mononucleated cells; n = 3 animals for each experimental group; each replicate was the pool of 2 mice; unpaired t test was used for comparison (*, P<0.05; **, P<0.01). (**D**) Representative FACS plot showing α7Sca1 GFP^+^ cells sorted from PBS (CTRL) or DT-injected mdx^ITGAM-DTR^ mice transplanted with SCs^GFP^ or FAPs^GFP^. (**E**) Graph reporting the percentage of α7Sca1 GFP^+^ cells sorted from PBS or DT-injected mdx^ITGAM-DTR^ mice transplanted with SCs^GFP^ or FAPs^GFP^; the percentage of α7Sca1 GFP^+^ cells is reported as relative to whole mononucleated cells; n = 3 animals for each experimental group; each replicate was the pool of 2 mice; unpaired t test was used for comparison (*, P<0.05). (**F, G**) Representative images of triple staining anti-perilipin (Plin) (red), anti-GFP (green), anti-caveolin (cyan) of TA cryosections of mdx^ITGAM-DTR^ mice not subjected to cell injection or transplanted with SCs^GFP^ and FAPs^GFP^ and injected with PBS (CTRL) or DT. Nuclei were counterstained with DAPI (white); n = 3 animals for each experimental group. Scale bar = 50 μm. (G) Graph reporting the quantification of total Plin^+^ cells and double Plin^+^/GFP^+^ cells, represented as stacked columns and reported as mean number per field (n) ±SEM (n = 3–5 biological replicates for each experimental group).

The transcriptome data suggested that α7Sca1 cells could derive from an adipogenic conversion of SCs in the dystrophic muscles as a consequence of the absence of signals derived from MΦs. To investigate this hypothesis, we performed a transplantation experiment in order to follow the fate of SCs and FAPs in mdx^ITGAM-DTR^ mice upon MΦ depletion. Specifically, as schematically reported in Fig 4A, SCs^GFP^ and FAPs^GFP^ cells were purified from mdx^GFP^ mice as α7^+^/Sca1^-^/Lin^-^ and Sca1^+^/α7^-^/Lin^-^, respectively (S6A Fig). SCs^GFP^ or FAPs^GFP^ cells were transplanted in GA and TA muscles of mdx^ITGAM-DTR^ mice, which were then injected with DT, or PBS as vehicle, following the standard schedule to yield a 15-day long period of MΦ depletion. The engraftment of transplanted SCs^GFP^ and FAPs^GFP^ cells was verified by performing anti-GFP immunofluorescence both in transplanted mdx^ITGAM-DTR^ mice (Fig 4B) and in non transplanted mdx^ITGAM-DTR^ mice upon PBS- or DT injection (S6B Fig). GFP staining further recapitulated the evidences reported above about the effect of MΦ depletion on endogenous SCs and FAPs: SCs decreased and FAPs increased upon MΦ depletion (Fig 4B). Moreover in DT-injected mice, SCs^GFP^ showed a non-canonical localization, interstitial instead of juxtaposed to muscle fibers, compared to the classical localization of SCs^GFP^ in control mice, suggesting drastic changes in SC identity induced by MΦ depletion.

We then performed a cytofluorimetric analysis to follow the appearance of α7Sca1 population and to check for the presence of α7Sca1^GFP^ cells both in SCs^GFP^ and FAPs^GFP^ transplanted mdx^ITGAM-DTR^ mice. This analysis clearly demonstrated that endogenous α7Sca1 cells were present both in SCs^GFP^ and FAPs^GFP^ transplanted mice at comparable extent upon DT injection, as was expected (Fig 4C); however, α7Sca1^GFP^ cells were detectable only in the animals transplanted with SCs^GFP^ and upon MΦ depletion (Fig 4D and 4E), but not in muscle transplanted with FAPs^GFP^ (Fig 4D and 4E). Moreover, we co-stained muscle cryosections from SCs^GFP^- and FAPs^GFP^- mdx^ITGAM-DTR^ transplanted mice or mdx^ITGAM-DTR^ non transplanted mice with GFP and Perilipin, an adipogenic marker, and we found the presence of double positive GFP-Plin cells only in SC-transplanted and DT-injected mdx^ITGAM-DTR^ mice (Fig 4F and 4G).

These results suggest that the α7Sca1 cells may originate from SCs undergoing an identity shift towards an adipogenic fate in a dystrophic MΦ-depleted environment.

### MΦs rescue SC differentiation defects via IL-10 signalling

**Fig 5:**
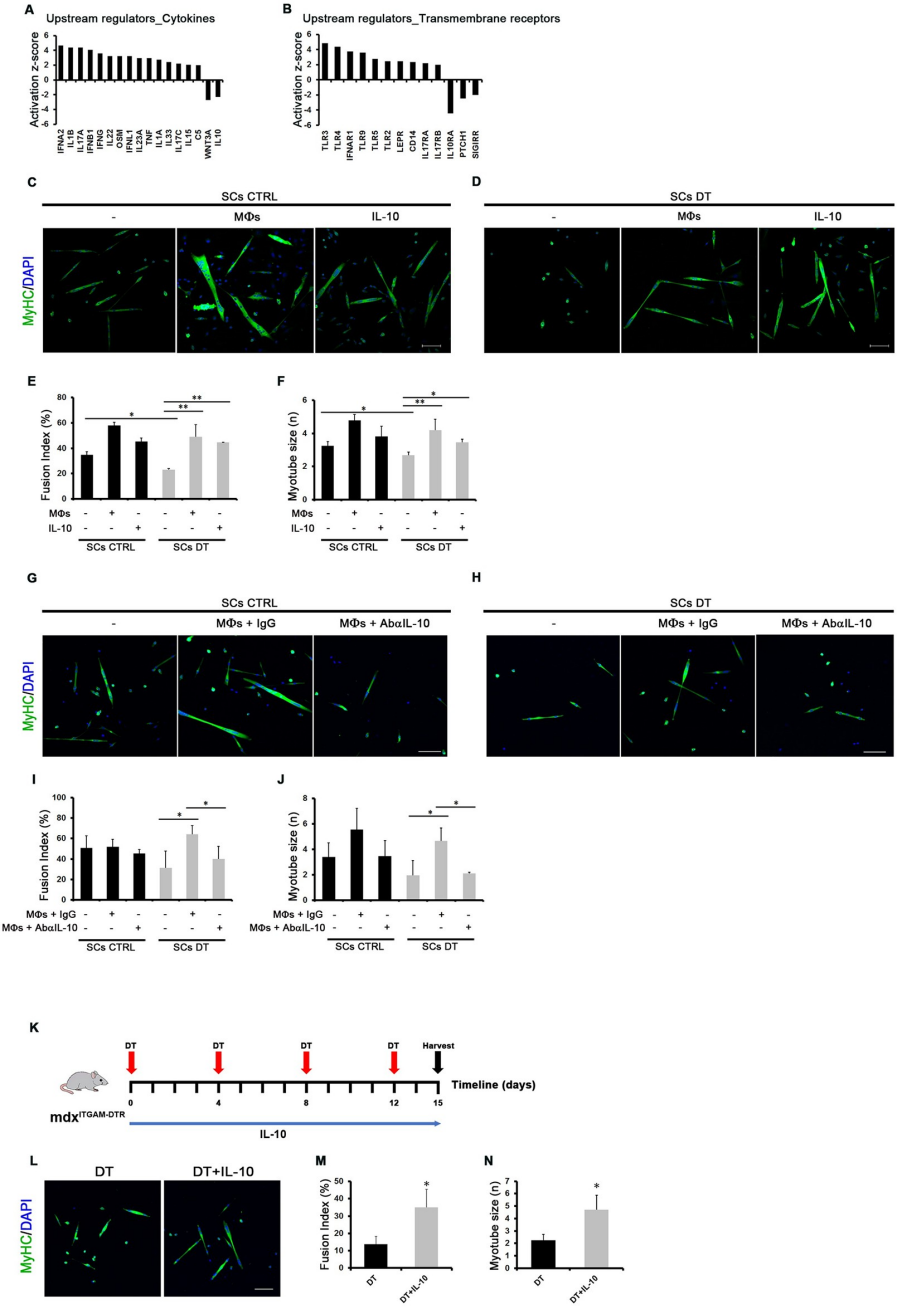
MΦs rescue SC differentiation defects via IL-10 signalling. (**A, B**) Ingenuity Pathway Analysis (IPA) of DEGs in SCs (DT vs CTRL) derived from mdx^ITGAM-DTR^ mice. The activation state of representative cytokines (A) and transmembrane receptors (B) is reported in the graphs with the relative Z-score. (**C, D**) Representative images of MyHC staining of differentiated SCs isolated from mdx^ITGAM-DTR^ mice injected with PBS (CTRL) or DT and co-cultured with MΦs purified from PBS (CTRL) injected mdx^ITGAM-DTR^ mice and seeded in an upper transwell or treated with recombinant cytokine IL-10. The cells were cultured in growth medium (GM) for 48 hours and then shifted in differentiation medium (DM) for further 48h. Both GM and DM medium contained recombinant IL-10. Nuclei were counterstained with DAPI (blue). Scale bar = 100 μm. (**E, F**) Graphs showing the quantification of two skeletal muscle differentiation parameters: fusion index (percentage of nuclei within myotubes: myotube = nuclei ≥ 2) and myotube size (mean of number of nuclei into myotubes). Data are represented as mean ± SEM (n = 4 independent experiments); one-way ANOVA was used for comparison (*, P<0.05; **, P<0.01). (**G, H**) Representative images of MyHC staining of differentiated SCs isolated from mdx^ITGAM-DTR^ mice injected with PBS (CTRL) or DT and co-cultured with MΦs purified from PBS (CTRL) injected mdx^ITGAM-DTR^ mice and seeded in an upper transwell. The co-culture was treated with IgG (MΦs+IgG) or an antibody anti-IL-10 (MΦs +AbαIL-10). The cells were co-cultured in growth medium (GM) for 48 hours and then shifted in differentiation medium (DM) for further 48h. Nuclei were counterstained with DAPI (blue). Scale bar = 100 μm. (**I, J**) Graphs showing the quantification of two skeletal muscle differentiation parameters: fusion index (percentage of nuclei within myotubes: myotube = nuclei ≥ 2) and myotube size (mean of number of nuclei into myotubes). Data are represented as mean ± SEM (n = 3 independent experiments); one-way ANOVA was used for comparison (*, P<0.05). **(K)** Experimental scheme for 15-day MΦ depletion combined with IL-10 treatment in mdx^ITGAM-DTR^ mice. DT was administered by intramuscular (im) injection every 4 days in 10 week-old mice, as described in S1B Fig. Either IL-10 or PBS, as control, were injected daily in TA and GA muscles of DT-injected mdx^ITGAM-DTR^ mice. **(L, M, N)** Representative images of MyHC staining of SCs isolated from mdx^ITGAM-DTR^ mice injected with DT and PBS (DT) or DT and IL-10 (DT+IL-10) and differentiated *ex-vivo*. The cells were cultured in growth medium for 48 hours and then shifted in differentiation medium for a further 48h. Nuclei were counterstained with DAPI (blue). Scale bar = 100 μm. In the graphs are reported the quantification of fusion index (percentage of nuclei within myotubes: myotube = nuclei ≥2) and myotube size (mean of number of nuclei into myotubes). Data are represented as mean ± SEM (n = 3 independent experiments); unpaired t test was used for comparison (*, P<0.05).

Ingenuity Pathway Analysis (IPA) of DEGs in SCs upon MΦ depletion was used to predict candidate upstream regulators that could be responsible for the observed gene expression changes and differentiative defects of SCs DT compared to SCs CTRL. Looking at specific categories of upstream regulators, this study suggested a deregulation of many signals presumably derived from MΦs in dystrophic muscles. Among these upstream regulators, IL-10 signalling was predicted as down-regulated, both in terms of cytokine and transmembrane receptor (Fig 5A and 5B), in the absence of MΦs as a known source of IL-10. Several papers highlighted an involvement of IL-10 in regeneration of dystrophic muscles [55–57]. To verify the direct effect of MΦs on SC differentiation defects in our model and to test the involvement of IL-10 signalling, we either co-cultured SCs CTRL or SCs DT with MΦs derived from mdx^ITGAM-DTR^ control mice or treated SCs cells with the cytokine IL-10 (Fig 5C and 5D). The measurement of muscle differentiation indexes demonstrated that the differentiation defects observed in SCs DT cultured *ex-vivo* could be rescued by the interplay with MΦs and by the IL-10 activity (Fig 5E and 5F).

We also confirmed the importance of the SCs- MΦs cross-talk via IL-10 signalling by using a blocking antibody anti-IL10 in the co-culture of SCs CTRL or SCs DT with MΦs (Fig 5G and 5H); indeed, the ability of MΦs to rescue the differentiation defects of SCs DT was prevented by blocking IL-10 derived from MΦs (Fig 5I and 5J).

Moreover, we evaluated the effects of daily intramuscular IL-10 injections in MΦ-depleted mdx^ITGAM-DTR^ mice, following the experimental scheme reported in Fig 5K. The muscle architecture appeared improved in terms of cell infiltration (S7A Fig) and the extent of fibrosis showed a slight, though not significant, decrease (S7B and S7C Fig); on the other hand, fat deposition of DT-injected mice was not altered by IL-10 injection (S7D Fig). The FACS analysis of muscle resident cell populations revealed no effects of IL-10 on SC number (S7E Fig) while the α7Sca1 cells were still present and showed the expected adipogenic phenotype (S7F Fig), indicating that IL-10 does not influence the decrease of SCs and the appearance of α7Sca1cells induced by MΦ-depletion. However, the *ex vivo* culture and differentiation of SCs demonstrated that the *in vivo* treatment of mdx^ITGAM-DTR^ mice with IL-10 counteracts the negative effects of MΦ depletion on SC differentiative potential (Fig 5L, 5M and 5N).

These data suggest that SC cell differentiation and fate choice are directly dependent on signals derived by MΦs and IL-10 plays a crucial role in mediating the SCs-MΦs cross-talk.

## Discussion

Our study provides new insight into the impact of the cross-talk between MΦs and SCs on cellular plasticity of SCs and, as a consequence, on the regenerative potential of dystrophic muscles. Our data are based on a new mouse model of transient and local depletion of MΦs in dystrophic mdx mice, achieved by DT injection (mdx^ITGAM-DTR^ mice). By using this mouse model, we demonstrate the need of MΦs to maintain SC cell fate and counteract muscle degeneration of dystrophic muscles. The results show that MΦ depletion determines an exacerbated dystrophic phenotype associated with increased fat deposition and fibrosis, reduction of SC number and the appearance of a cell population with adipocyte-like phenotype. Interestingly, the SC phenotype was deeply affected, both in terms of self-renewal and differentiation, and in terms of cell fate choice. In fact, MΦ depletion not only caused reduced proliferation and precocious myogenic differentiation of SCs associated with defects in the formation of mature myofibers, but also promoted a shift to adipogenic differentiation which suggested that MΦs can orchestrate the SC cell fate during regeneration of dystrophic muscles.

Thus, beside the cell autonomous commitment of SCs, other cell populations recruited in regenerating muscles affect the SC fate in a non-cell autonomous manner.

Specifically, in our dystrophic model of MΦ depletion SCs appear to move from the classic position under the basal lamina toward an interstitial localization and they acquire an adipogenic expression pattern (Figs 1C, 2A, 2G and 4B), suggesting a loss of identity of SCs culminating into mesenchymal-adipogenic conversion. SCs are known to hold mesenchymal plasticity that enables them to commit towards a mesenchymal differentiation program as an alternative to myogenesis [50,58] and recent papers postulated the acquisition of mesenchymal-like characteristics and fibrogenic conversion of SCs linked to increased TGF-β signaling in advanced stages of dystrophy [11,12]. A similar process could also lead to adipogenesis. The alternative cell fate, muscular or adipogenic, of SCs has been well characterized [49,52,53,58]; however our data provide the first demonstration that the adipogenic choice of SCs is dependent on SCs-MΦs cross-talk. In particular, co-culture experiments and genome-wide expression profiles of SCs freshly sorted from MΦ-depleted dystrophic muscles showed a strong reduction in the expression level of MyoD and Myf5 (Fig 2C), whose activity is essential not only to induce muscle differentiation but also to repress the alternative adipogenic cell fate of muscle progenitor cells [49,50]. Furthermore, in MΦ-depleted dystrophic muscles, FACS analysis identified a novel cell population referred to as α7Sca1 cells since they are positive for both α7int and Sca1, which are surface markers specifically expressed by SCs and mesenchymal precursors (FAPs), respectively. However, it’s worth noting that a recent paper demonstrated that SCs are not the only muscle cells expressing α7-integrin [59]. The *ex vivo* culture and the transcriptome of α7Sca1 cells revealed an adipogenic-like phenotype. However, the comparison of α7Sca1 transcriptome with published datasets of brown and white adipose tissue (GSE56367) [54] confirmed that α7Sca1 cells have a substantially different expression pattern (S7G Fig), supporting the hypothesis that these cells represent a novel pathological muscle-specific cell population possibly contributing to the observed fat accumulation and exacerbation of dystrophic phenotype. A detailed study is required to strictly verify this hypothesis. Transplantation in mdx^ITGAM-DTR^ mice of tracing SCs^GFP^ or FAPs^GFP^ derived from mdx^GFP^ mice revealed that α7Sca1 cells originate from SCs that acquire adipogenic markers in a MΦ-depleted dystrophic environment. However, independently of the detrimental effects of adipocyte-like α7Sca1 cells, the conversion of SCs to α7Sca1 cells exert negative effects on the dystrophic phenotype as SCs are exhaustible and indispensable for proper muscle regeneration.

Overall, our data suggest that MΦs control the cell fate and, in turn, the regenerative potential of SCs in dystrophic muscles. Specifically, transient MΦ depletion in a dystrophic mouse model determines loss of identity and adipogenic conversion of SCs, thus resulting in the exhaustion of the stem cell pool and exacerbated dystrophic phenotype.

A caveat to the interpretation of the data is that the effects of MΦ depletion on muscle regeneration could also be due to a different balance of inflammatory components in the muscle environment generated by MΦ depletion. For instance, we measured an increase in neutrophil number upon MΦ depletion, an increase potentially contributing to exacerbate the dystrophic phenotype [60]. However, the co-culture experiments demonstrated that the differentiation defects of SCs are a direct consequence of the absence of signals derived from MΦs. Independently from a discrimination between a direct or indirect effect of MΦ activity, clearly the drastic imbalance of the inflammatory populations in the dystrophic muscle niche upon MΦ depletion determines severe effects on regeneration, highlighting a crucial role of the interplay between SCs and MΦs to limit the degeneration and extend the regenerative potential of dystrophic muscles.

These findings and other studies confirm the point that, besides the previously proven beneficial effects of anti-inflammatory drugs on the dystrophic phenotype, the use of broad, non- specific anti-inflammatory approaches could be even detrimental to muscle regeneration by interfering with the pro-regenerative cross-talk between the immune system and muscle resident cells [55,61]. Although the exact mechanism by which MΦs control the cell fate of SCs remains to be understood, the activity of MΦs is very likely to be involved in defining SCs cell identity. SCs derived from MΦ-depleted muscles and cultured *ex vivo* showed differentiation defects in terms of myotube formation, suggesting an imprinted memory resulting from environmental cues. However, this imprinting appears to be reversible; in fact, it is partially rescued by *ex vivo* co-culture with MΦs or by treatment with recombinant IL-10 cytokine. Previously, several reports suggested a critical role of IL-10 in the regeneration of dystrophic muscles [55–57]. In our study, IL-10 pathway was predicted as being inhibited in SCs DT and *ex vivo* experiments confirmed that IL-10 signalling is involved in modulating proper SC differentiation; indeed, the differentiation defects of SCs DT are partially rescued by IL-10, suggesting that SC activity could be dependent on an appropriate regulation of the IL-10 pathway. The characterization of MΦ-mediated signalling pathways involved in modulating self-renewal, commitment and differentiation of SCs could be therapeutically exploited to increase the regenerative potential of SCs. Considering the reported results, therapeutic approaches targeting MΦs in dystrophic muscles should be accurately evaluated in terms of their effect on SCs-MΦs cross-talk and on SC cell fate choice.

## Materials and methods

### Mouse models

Housing, experimental protocols and procedures were conducted following guidelines of the institutional Animal Research Ethical Committee at Fondazione Santa Lucia (FSL) according to the Italian Ministry of Health and complied with the NIH Guide for the Care and Use of Laboratory Animals.

mdx mice (C57BL/10ScSn-DMD^mdx^/J) were purchased by Charles River, Italy. Both male and female mice at 10–12 weeks of age were used for the experiments described and they were randomly assigned into experimental groups, including the same number of male and female mice in each group.

mdx^ITGAM-DTR^ mice were generated in our laboratory by crossing mdx mice with ITGAM-DTR mice (B6.FVB-TG-ITGAM-DTR/EGFP-34LAN/J) obtained from The Jackson Laboratory, following the breeding strategy reported in S1A Fig. For each generation we performed genotyping analysis to test the presence of ITGAM transgene by standard PCR (See S1 Table for primer sequences). Homozygous mdx^ITGAM-DTR^ were selected by qPCR (See S1 Table for primer sequences).

Homozygous mdx^ITGAM-DTR^, both male and female mice at 10–12 weeks of age were used for the experiments described and they were randomly assigned into experimental groups, including the same number of male and female mice in each group.

mdx^GFP^ mice were generated in the lab of M. Bouché (Sapienza University of Rome) by breeding mdx mice, C57BL/10ScSn-Dmdmdx/J, with GFP expressing mice (C57BL/6-Tg (UBC-GFP)30Scha/J, The Jackson Laboratory), where the expression of the GFP protein is under the control of the housekeeping Ubiquitin C promoter. Those mice which resulted homozygous for the mutant dystrophin gene, by gene sequencing, and positive for GFP, by tail staining, were bred to maintain the colony. GFP expression was verified in the offspring, before use.

mdx^ITGAM-DTR^ and mdx mice (as control reference) were treated with Diphtheria Toxin (DT; Sigma-Aldrich Cat#: D0564) in order to deplete macrophages. DT was dissolved at 2.5 ng/ml in PBS and injected in TA (10 μl, single injection) and in GA (10 μl, two injections) muscles. DT injection has been repeated every 4 days. 15 days after the first DT injection, the animals were sacrificed by cervical dislocation. Control mice were injected with the same volume of the vehicle PBS following the same schedule. A schematic description of experimental schedule is reported in S1B Fig. TA muscles were collected for histological analyses, RNA and protein extraction. GA muscles were used for isolation of primary cells, as described below. For the experimental scheme in S1B Fig, Figs 4A and 5K, we modified a mice picture derived from a published paper [62].

IL-10 was dissolved in PBS and injected daily (0.25 μg/day) in TA (10 μl, single injection) and in GA (10 μl, two injections) muscles. Control mice were injected with the same volume of the vehicle PBS following the experimental schedule reported in Fig 5K. When both DT and IL-10 were scheduled, one of the two injections was performed 8 hours later.

### FACS isolation and culture

Satellite cells (SCs), fibroadipogenic progenitors (FAPs), macrophages (MΦs), and α7Sca1 cells were purified by FACS and cultured *ex vivo*, or used for cell transplantation experiments.

Hind limb muscles isolated from experimental mice were finely minced with sharp scissors and used for primary cell isolation. Specifically, minced GA muscles were digested in an enzymatic cocktail containing Collagenase A (2 μg/mL), Dispase I (2.4 U/mL), DNase I (10 ng/mL) in PBS supplemented with Ca^2+^ and Mg^2+^, at 37 °C under gentle agitation, for 1 hour. The digestion solution was diluted by adding HBSS supplemented with 0.2% BSA and then the samples were filtered through 40 μm cell strainer (Falcon). Pelletted cells were then resuspended in HBSS + 0.2% BSA and stained with the following antibodies: 1:50 CD31-Pacific blue (Life Technologies Cat#: RM5228, clone # 390), 1:50 CD45-Pacific blue (eBioscience, Cat#: MCD4528, clone #30-F11), Ter119-Pacific blue (eBioscience, Cat#: 48-5921-82, clone TER-119), 1:200 CD11b-PECy7 (BD Biosciences, cat#552850, clone M1/70), 1:50 F480-PE (ThermoFisher Cat#: 14-4801-81, clone BM8), 1:500 α7-integrin-APC-647 (Alexa-Fluor 647; AbLab Cat#: AB10RS24MW215, clone R2F2), 1:50 Sca1-FITC (eBioscience, Cat#: 11-5981-81 clone D7), 1:200 Gr1-APC-e780 (eBioscience, BMS47-5931-80). Resuspended cells were also labelled with 1:200 LIVE/DEAD-Pacific orange dye (Fixable Aqua Dead Cell Stain Kit, for 405 nm excitation) in order to discard dead cells at the moment of FACS analysis. Briefly, primary cells were purified by Beckman Coulter MoFlo High Speed Sorter (Software Summit V4.3.01) and analyzed using FloJo software (Software FlowJo, LLC,Treestar, CA, USA, v. 10.4.2) as follows: cells were first separated in Lineage (Lin: CD31, CD45, Ter119) positive and negative cells. SCs were isolated as Lin^-^/Sca1^-^ /α7^+^ cells; FAPs were isolated as Lin^-^/Sca1^+^/α7^-^ cells; MΦs were isolated as Lin^+^/Sca1^-^/α7^-^/CD11b^+^/F480^+^ cells; neutrophils were gated as Lin^+^/Sca1^-^/α7^-^/CD11b^+^/Gr1^+^ cells.

SCs were seeded on gelatin-coated plates (Stem Cell Technologies) at low density (2500 cells/cm^2^) and cultured in SC growth medium containing high-glucose (4500 mg/L) DMEM-GlutaMAX (Gibco) supplemented with Penicillin-Streptomycin (ThermoFisher), 20% (v/v) horse serum (Gibco), 10% (v/v) fetal bovine serum (Gibco), 1% (v/v) Chicken Embryo Extract (Seralab CE-650-J), for 4 days, with a change of medium after 2 days. Afterwards, SCs were shifted in low-serum medium (differentiation medium) containing high-glucose (4500 mg/L) DMEM-GlutaMAX (Gibco) supplemented with Penicillin-Streptomycin (ThermoFisher), 2% (v/v) horse serum (Gibco), 0.5% (v/v) Chicken Embryo Extract (Seralab CE-650-J) for 2 days.

For high-density differentiation assay freshly sorted SCs were first seeded at low density (2500 cells/cm^2^) in growth medium for 4 days. The cells were then detached, pooled and plated at high density (60000 cells/ cm^2^) in growth medium for a few hours and once adhered, the cells were shifted in differentiation medium for 2 days.

For co-culture experiments MΦs from control (PBS-injected) mice were seeded in an upper transwell with 1.0 μm pore (Falcon, #353104; Sarstedt) containing high-glucose (4500 mg/L) DMEM-GlutaMAX (Gibco) supplemented with Penicillin-Streptomycin (ThermoFisher), 20% (v/v) horse serum (Gibco), 10% (v/v) fetal bovine serum (Gibco), 1% (v/v) Chicken Embryo Extract (Seralab CE-650-J) for 2 days and then the transwell were transferred on seeded SCs and maintained in growth medium for 2 additional days before the shift in differentiation medium for 2 days. SCs and MΦs were co-cultured with a ratio SCs: MΦs = 1:3. Where described growth medium and differentiation medium were supplemented with 2.5 μg/ml of IgG (S2 Table) or an antibody anti IL-10 (S2 Table).

Where described, SC growth and differentiation media were supplemented with 50 ng/ml of recombinant IL-10 (Peprotech Cat#: 210–10), dissolved in 0.1% BSA. Media of control cells were supplemented with 0.1% BSA. Specifically IL-10 was added to the growth medium 2 days following plating and then replaced at the moment of the shift in differentiation medium (day 4). The cells were then maintained in differentiation medium for 2 days. Cultured cells were fixed in 4% Paraformaldehyde (BDH).

α7Sca1 cells were seeded on gelatin-coated dishes either in SC growth medium or in FAP growth medium (Cyto-Grow; Resnova TGM-9001-A), for 2 days. Cultured cells were then fixed in 4% Paraformaldehyde (BDH).

### Histological analysis and immunohistochemistry

TA muscles from mdx^ITGAM-DTR^ and mdx mice were isolated, embedded in OCT (Tissue-Tek) and frozen in liquid nitrogen-cooled isopentane (Sigma-Aldrich). Embedded muscles were then sectioned into 8 μm sections on a Leica cryostat (Leica CM1850UV).

For hematoxylin-eosin staining, muscle sections were fixed with 4% paraformaldehyde (BDH) and then stained with hematoxylin and eosin (Hematoxilyn: Sigma-Aldrich Cat#: HHS32; Eosin: Sigma-Aldrich Cat#: HT110332) dyes, followed by sequential dehydratation in 75%, 90%, 100% ethanol (Sigma-Aldrich) and xylene (Sigma-Aldrich) and mounted with EUKITT (Sigma-Aldrich Cat#: 03989).

For Oil Red O (ORO) staining, muscle sections and cells were fixed with 4% PFA (BDH) for 1 h or 10’ respectively, washed, dehydrated in 60% Isopropanol (Merck), stained with ORO dye (Sigma-Aldrich Cat#: O0625, Sigma-Aldrich), counterstained with Hematoxilin and then washed. Muscle sections were mounted with 10% glycerol (Sigma-Aldrich).

For Sirius Red, muscle sections were fixed with Bouin’s solution (Sigma-Aldrich Cat#: HT10132) for 1 h, washed and then stained with Picro-Sirius Red dye (Sigma-Aldrich Cat#: CI 35780) for 1h, followed by sequential dehydration in 90%, 100% ethanol and xylene and then mounted with EUKITT (Sigma-Aldrich Cat#: 03989).

For immunofluorescence, muscle sections were fixed with cold acetone (Sigma-Aldrich) for 1’, air-dried and washed. PBS (Gibco) was used for all washing steps both for muscle sections and cultured cells. After blocking with 4% IgG-free BSA (Jackson Lab, 105696) in PBS for 45’, muscle slides were incubated overnight with primary antibodies, at indicated dilutions, at 4 °C. Samples were then washed with 1% IgG-free BSA and then incubated with an appropriate secondary antibody for 1 h, at room temperature. The nuclei were counterstained with 4’,6 diamidino-2-phenylindole (DAPI; ThermoFisher Cat#: D1306) and then washed three times.

Muscle cryosections were stained with the following antibodies at indicated dilutions: 1: 20 anti-eMyHC (DSHB: Developmental Studies Hybridoma Bank, clone F1.652); 1:400 anti-Laminin (Sigma-Aldrich Cat#: L9393); 1:200 anti-Perilipin (Sigma-Aldrich Cat#: P1873): 1:400 anti-Caveolin-3 (BD Transduction Laboratories Cat#: 610420); 1:20 anti-Pax7 (DSHB: Developmental Studies Hybridoma Bank; PAX7-s); 1:150 anti-F480 (Bio-Rad Cat#: MCA497G); 1:200 anti-Col1 (AbCam Cat#: ab6308); 1:400 anti-GFP (AbCam Cat#: ab6556); 1:200 anti-GFP (SantaCruz Cat#: 9996); 1:3 anti-Myogenin (DSHB: Developmental Studies Hybridoma Bank, clone F5D); 1:500 anti-A2Laminin (Alexis, ALX-804-190-C100).

Cells were fixed with 4% PFA for 10’, rinsed with 50 mM Glycine (BDH) for 10’ and permeabilized with 0.1% Triton (Sigma-Aldrich) for 10’. Cells were then blocked with 4% IgG-free BSA for 45’. Primary antibodies were applied to samples with indicated dilution factors and then kept at 4 °C overnight. Samples were then washed with 1% IgG-free BSA and then incubated with an appropriate secondary antibody for 1 h, at room temperature. The nuclei were counterstained with DAPI (ThermoFisher Cat#: D1306) and washed. SCs were stained with the following primary antibody at indicated dilutions: 1:20 anti-MyHC (MF20, DSHB: Developmental Studies Hybridoma Bank). For cell proliferation analysis, SCs were cultured in GM for 2 days and then EdU were added for 4 h in fresh GM before the fixation. Cells were then stained using Click-iT EdU Alexa Fluor Imaging Kit (Thermofisher Cat# C10337) and 4′,6-diamidino-2 phenylindole (DAPI). Proliferating cells were counted checking the co-localization of DAPI and EdU positivity.

Secondary antibodies: AlexaFluor 647 Goat anti-Rat IgG (H+L) (Life Technologies A21247); Alexa Fluor 594 Goat anti mouse (H+L) (Life Technologies A150116); Alexa Fluor 488 Goat anti mouse (H+L) (Life Technologies A32723); Alexa Fluor 488 Goat anti rabbit (H+L) (Life Technologies A-11034); Alexa Fluor 594 Goat anti rabbit (H+L) (Life Technologies A-11012).

Image acquisition was performed by Nikon Upright fluorescent microscope (Nikon DXM1200F), Leica DM IL (Camera “Leica DFC420 C” Software:Leica Application Suite v2.6.0 R1), Nikon ECLIPSE TE2000-E (Camera: Nikon D-Eclipse C1 Si, Software: NIS elements v4.20).

All quantifications on histology and immunofluorescence analyses were performed using ImageJ software version 1.52e (https://imagej.nih.gov/ij/download.html). Specifically, the quantification of ORO and Sirius Red positive area was performed on 7–9 fields/section of TA muscle, corresponding to approximatively 80%-90% of whole muscle cross section.

### Cell transplantation experiments

For cell transplantation experiments, SCs^GFP^ and FAPs^GFP^ isolated from 8–12 week-old mdx^GFP^ mice, by FACS as described above. Then the cells were injected in TA and GA muscles of 10 week-old mdx^ITGAM-DTR^. Specifically 4x10^4^ sorted SCs^GFP^ or FAPs^GFP^ were transplanted into left or right TA muscles, respectively, few hours before inducing 2-week long MΦ depletion by DT injection, as described above. Control mice were injected with the vehicle PBS after cell transplantation. PBS/DT injection were repeated every 4 days and 15 days after cell transplantation and first PBS/DT injection, the mice were sacrificed. TA muscles were collected for histological and immunohistochemistry analyses; GA muscles were used for analysis of primary cell populations by FACS.

### Expression analysis by RT-qPCR

Total RNA from whole muscles was obtained by homogenizing TA muscles with a tissue homogenizer (Tissue Ruptor Qiagen Cat #: 990890) in TriReagent (Sigma-Aldrich Cat#: T9424). The RNA extraction was performed following TriReagent manufacturer’s protocol.

Total RNA from freshly sorted cells was extracted with RNeasy Plus Micro kit (Qiagen Cat#: 74004) or RNeasy Plus Mini kit (Qiagen Cat#: 74104) following manufacturer’s protocol. RNA was quantified with NanoDrop (Thermo Scientific NanoDrop 2000C).

For mRNA analysis, 50–500 ng of RNA was retrotranscribed with random primers and RT kit (Thermo Fisher Cat#: 8080234). qPCR analysis was performed with SYBR Green Master Mix (Eppendorf, Primer Design Cat#: T PRECPLUS-R-SY) and using primer pairs manually designed with Primer3 (primer3.ut.ee). The reactions were run on 7900HTABI prism PCR machine (Applied Biosystems). All the murine expression primers used in this study span an exon-exon junction. The sequences of murine expression oligonucleotides for qPCR are listed in S1 Table.

All values were obtained in duplicate or triplicate and the analysis of output values was made using standard ΔΔCt method.

### RNA-Sequencing: Libraries, sequencing and analysis

RNA-Seq was performed on biological duplicates of freshly isolated SCs (3.5x10^5^ and 4x10^5^ cells for PBS condition; 1.9x10^5^ and 2,5x10^5^ cells for DT condition) and α7Sca1 cells (3.7x10^4^ and 8x10^4^ cells for DT condition), using the SMART-seq2 protocol [63] with minor modifications. Each biological sample derived from the pool of GA muscles of two mice. Briefly, 5 ng of total RNA extracted with Qiagen RNeasy micro kit (Qiagen Cat#: 74004) was retro transcribed with oligo dT and LNA-containing template-switching oligo (TSO). The resulting cDNA was pre-amplified, purified and tagmented with Tn5 transposase produced in-house using a described protocol [64]. cDNA fragments generated after tagmentation were gap-repaired, tagged and enriched by PCR. The final cDNA library was purified with SPRI beads (Beckman-Coulter Agencourt AMPure XP, Cat#: A63881), measured with a Qubit fluorometer and sequenced on a Hiseq2000 Illumina platform.

HTS-flow framework [65] was used to perform the alignment of raw reads (50 bp single-end reads) determining absolute and differential gene expression. Raw reads (FastQ files) were aligned to the Mus Musculus reference genome (NCBI37/mm9) using topHat (version 2.0.6); the absolute expression is quantified by calculating RPKMs (reads per million library size, per kb of transcript size); differential expression is determined using the DESeq2 Bioconductor library, with standard parameters (adjusted p-value lower than 0.05 and a maximum RPKM expression greater than 0.25). Gene functional enrichment was performed by DAVID tool (Version 6.8) [66,67] (https://david.ncifcrf.gov/). The analysis of Gene Ontology (GO) terms was restricted to the terms with adjusted P-value lower than 0.05. The prediction of deregulated pathways and upstream regulators was performed by software IPA (Ingenuity Pathway Analysis; https://www.qiagenbioinformatics.com/products/ingenuity-pathway-analysis/).

### Statistical analysis

All quantitative data are presented as mean ± standard error mean (SEM), represented as error bars. The statistical significance was assessed by using either a two-tailed, unpaired Student’s t test to calculate differences between two groups or one-way ANOVA with post-hoc test for multiple comparisons (http://astatsa.com/OneWay_Anova_with_TukeyHSD/). P values ≤ 0.05 were considered as statistically significant and are indicated in each figure legend; p values > 0.05 were considered as statistically not significant (n.s). The number of animals or samples for each experiment is indicated in the relative figure legends.

### Data and software availability

RNA-seq data have been deposited in a public database (GEO: accession number GSE134770; https://www.ncbi.nlm.nih.gov/geo/query/acc.cgi?acc=GSE134770).

### Ethics statement

This study included vertebrate animals, specifically mouse models.

Housing, experimental protocols and procedures were conducted following guidelines of the institutional Animal Research Ethical Committee at Fondazione Santa Lucia (FSL) according to the Italian Ministry of Health and complied with the NIH Guide for the Care and Use of Laboratory Animals. The relative obtained approval numbers are 82/2014 PR and 340/2018PR.

## Supporting information

S1 Fig MΦ depletion in mdx^ITGAM-DTR^ mouse model. (**A**) Breeding scheme followed for the generation of mdx^ITGAM-DTR^ mice, by crossing mdx mice and ITGAM-DTR mice. (**B**) Experimental scheme for 15-day MΦ depletion in mdx^ITGAM-DTR^ mice. Either DT or PBS as vehicle, was administered by intramuscular (im) injection every 4 days in 10 week-old mice. DT was injected at 1 ng/g body weight, one injection in Tibialis Anterior (TA) muscles and two injections in Gastrocnemius (GA) muscles, 4 injections total; the mice were sacrificed 15 days (d15) after the first injection. **(C)** Flow cytometry gating strategy used to purify SCs, FAPs and MΦs from hind limb GA muscles of mdx^ITGAM-DTR^ mice. FSC x SSC gating was used to obtain mononuclear cells on the basis of size and granularity. Live/dead (LD) Aqua marker was used to identify live cells (Aqua negative cells). Staining with anti-hematopoietic lineage (Lin) antibodies, anti- CD31, CD45 and Ter-119 was performed to separate Lin^+^ from Lin^-^. From Lin^-^ subpopulation, SCs was purified as α7integrin^+^ (APC), which are negative for Sca1 (FITC). FAPs was identified as Sca1^+^ (FITC) α7integrin^-^ cells. From Lin^+^ subpopulation, macrophages, which are CD31^-^, CD45^+^ and Ter-119^-^ was identified as CD11b^+^ (PC7) and F4/80^+^ (PE) double positive cells. (**D-E**) FACS plot showing MΦ population in mdx^ITGAM-DTR^ mice im injected with PBS (CTRL) or DT. The mice were sacrificed 15 days after the first intramuscular (im) injection of DT (1 ng/g body weight), one injection in TA muscles and two injections in GA muscles; the DT injection has been repeated every 4 days (see Experimental scheme in S1B Fig. MΦs were sorted from TA and GA muscles as Lin^+^/CD11b^+^/F4/80^+^ cells; in the graph is reported the percentage of MΦs expressed as relative to whole mononucleated cells; values are mean ± SEM; n = 6 animals for each group; unpaired t test was used for comparison (**, P<0.01;). (**F**) Graph showing MΦ depletion in mdx^ITGAM-DTR^ mice at d3, d7, d11 along the schedule of DT injection reported in S1B Fig. MΦs were sorted from TA and GA muscles as CD11b^+^/F4/80^+^ cells from Lin^+^ subpopulation; in the graph is reported the percentage of MΦs expressed as relative to whole mononucleated cells; DT samples are compared to PBS-injected mice (CTRL) sacrificed at d11; values are mean ± SEM; n = 3 animals for each group; unpaired t test relative to CTRL was used for comparison of each DT sample (**, P<0.01; ***, P<0.001). (**G**) Representative images of double staining anti-caveolin (red) and anti-F4/80 (cyan) of TA cryosections of mdx^ITGAM-DTR^ mice injected with PBS or DT, as described for the S1B and S1D Fig. Nuclei were counterstained with DAPI (white); n = 6 animals for each group. Scale bar = 100 μm. (**H-I**) FACS plot showing neutrophils in mdx^ITGAM-DTR^ mice im injected with PBS (CTRL) or DT as described for the S1B and S1D Fig. Neutrophils were sorted from TA and GA muscles as CD11b^+^/Ly6G^+^ (GR1) cells. In the graph is reported the percentage of neutrophils expressed as relative to whole mononucleated cells; values are mean ± SEM; n = 3 animals for each group; unpaired t test was used for comparison (**, P<0.01). (**J-K**) FACS plot showing MΦ population in mdx mice im injected with PBS (CTRL) or DT (DT), as described in S1B Fig. MΦs were sorted from GA muscles as Lin^+^/CD11b^+^/F4/80^+^ cells. In the graph is reported the percentage of MΦs expressed as relative to whole mononucleated cells; values are mean ± SEM; n = 4 animals for each group; unpaired t test was used for comparison (n.s. = not significant).
(TIF)

S2 Fig MΦ depletion compromises muscle regeneration in dystrophic mice. (**A**) Representative images of Hematoxilin/Eosin staining on cryosections of TA muscle derived from mdx^ITGAM-DTR^ mice injected with PBS or DT. Scale bar = 200 μm. (**B**) Mean Cross Sectional Area (CSA) of muscle fibers, measured on laminin-stained cryosections. Values are mean ± SEM (n = 3 animals for each experimental group); unpaired t test was used for comparison (**, P<0.01). (**C**) Frequency distribution of muscle fibers CSA of mdx^ITGAM-DTR^ mice injected with PBS or DT. Values are mean ± SEM (n = 3 animals for each experimental group); unpaired t test was used for comparison (*, P<0.05; **, P<0.01; ***, P<0.001). (**D, E**) Representative images of double staining anti-laminin (cyan) and anti-eMyHC (red) of TA cryosections of mdx^ITGAM-DTR^ mice injected with PBS or DT. Nuclei were counterstained with DAPI (white); n = 3 animals for each experimental group. Scale bar = 100 μm. In the graph is reported the percentage of eMyHC positive myofibers relative to total cells; values are mean ± SEM; n = 3 animals for each experimental group; unpaired t test was used for comparison (***, P<0.001). (**F, G**) In the graphs are reported the total number of myofibers per section (p = 0.07) (**F**) and the number of myonuclei/150 eMyHC^+^ myofibers, measured on laminin-eMyHC co-stained cryosections. (**G**) Values are mean ± SEM (n = 3 animals for each experimental group); unpaired t test was used for comparison (*, P<0.05). (**H, I**) Expression analysis of muscle markers by qRT-PCR on whole muscle (TA) derived from mdx^ITGAM-DTR^ mice injected with PBS or DT. Expression data are reported as relative to housekeeping gene TBP, and represented as mean ±SEM (n = 3–7 biological replicates for each experimental group); unpaired t test was used for comparison (*, P<0.05; **, P<0.01).
(TIF)

S3 Fig MΦ depletion increases fibrosis and fat deposition. (**A-B**) Representative images of Sirius red staining on TA cryosections derived from mdx^ITGAM-DTR^ mice injected with vehicle (CTRL) or DT as described in S1B Fig. In the graph is reported the percentage of fibrotic area; values are mean ± SEM; n = 7 animals for each group; unpaired t test was used for comparison (**, P<0.01). Scale bar = 200 μm. (**C, D**) Representative images of Oil Red O staining on TA cryosections of mdx^ITGAM-DTR^ mice injected with PBS (CTRL) or DT; n = 7 animals for each group. In the graph is reported the percentage of Oil Red O positive area; values are mean ± SEM; n = 7 animals for each group; unpaired t test was used for comparison (***, P<0.001). Scale bar = 100 μm. (**E**) Representative images of double staining anti caveolin (green) and anti perilipin (red) of TA cryosections of mdx^ITGAM-DTR^ mice injected with PBS (CTRL) or DT. Nuclei were counterstained with DAPI (white); n = 3 animals for each group. Scale bar = 50 μm. (**F**) Expression analysis of fibrosis and adipogenesis markers by qRT-PCR on whole muscle (TA) derived from mdx^ITGAM-DTR^ mice injected with PBS (CTRL) or DT. Expression data are reported as relative to housekeeping gene TBP, and represented as mean ± SEM (n = 6 biological replicates for each experimental group); unpaired t test was used for comparison (*, P<0.05; **, P<0.01; ***, P<0.001).
(TIF)

S4 Fig DT injection does not affect muscle regeneration in mdx mice. (**A, B**) Representative images of Hematoxilin/Eosin staining on TA cryosections derived from mdx mice injected with PBS or DT. In the graph is reported the mean of the Cross Sectional Area (CSA) of muscle fibers, measured on laminin-stained TA cryosections of mdx mice. Values are mean ± SEM (n = 3 animals for each experimental group); unpaired t test was used for comparison (n.s. = not significant). Scale bar = 200 μm. (**C, D**) Representative images of Sirius red staining on TA cryosections derived from mdx mice injected with vehicle (CTRL) or DT as described in S1B Fig. In the graph is reported the percentage of Sirius Red positive area; values are mean ± SEM (n = 4 PBS and 5 DT animals for each experimental group); unpaired t test was used for comparison (n.s. = not significant). Scale bar = 200 μm.
(TIF)

S5 Fig Muscle cell populations in DT-injected mdx mice. Comparative GO analysis of SCs and α7Sca1 cells isolated from mdx^ITGAM-DTR^ mice. (**A, B, C**) FACS plot showing SCs, FAPs and α7Sca1 cell populations isolated from mdx mice im injected with PBS (CTRL) or DT (DT). Cells isolated from GA muscles were first separated into hematopoietic lineage positive (Lin+) and hematopoietic lineage negative (Lin-) (Lin: CD45, CD31 and Ter119) cells. SCs and FAPs were sorted as Lin^-^/α7int^+^/Sca1^-^ and Lin^-^/Sca1^+^/α7int^-^/ cells, respectively. α7Sca1 cells (α7^+^/ Sca1^+^/Lin^-^) was not detectable in mdx mice. In the graphs are reported the percentage of SCs and FAPs expressed as relative to whole mononucleated cells; values are mean ± SEM; n = 4 animals for each group; unpaired t test was used for comparison (n.s. = not significant). (**D, E, F**) Selected representative GO biological processes in Cluster I (C), Cluster II (D) and Cluster III, as indicated in the heat map in Fig 5K and identified by DAVID 6.8. The graph displays for each GO term the obtained p value (expressed as −log10) on the x axis and the number of genes included (count), on the y axis. (**G**) Hierarchical clustering comparing the expression patterns of SCs, FAPs, α7Sca1 cells, BAT (brown adipose tissue) and WAT (white adipose tissue). BAT and WAT RNA-Seq samples (in triplicate) were downloaded from the GSE56367 GEO series; raw reads were filtered and aligned to mm9 similarly to the samples generated by us (SCs, FAPs, α7Sca1 cells), and absolute RPKMs for 21K genes were determined. Absolute RPKMs were normalized altogether.
(TIF)

S6 Fig Transplantation of SCs^GFP^ and FAPs^GFP^ derived from mdx mice in mdx^ITGAM-DTR^ mice. (**A**) Flow cytometry gating strategy used to purify SCs^GFP^ and FAPs^GFP^ from hind limb GA muscles of mdx^GFP^ mice. FSC x SSC gating was used to obtain mononuclear cells on the basis of size and granularity. GFP^+^ cells were stained with and with lineage-specific (Lin) antibodies CD31, CD45 and Ter-119 to separate Lin^+^ from Lin^-^. From GFP^+^/Lin^-^ subpopulation, SCs were purified as α7integrin^+^ (APC), which are negative for Sca1 (FITC). FAPs were identified as Sca1^+^ (FITC) α7integrin^-^ cells. (**B**) Representative images of double staining anti-caveolin (red) and anti-GFP (green) of TA cryosections of mdx^ITGAM-DTR^ mice no-cell transplanted and injected with PBS (CTRL) or DT. Nuclei were counterstained with DAPI (white); n = 3 animals for each experimental group. Scale bar = 50 μm.
(TIF)

S7 Fig IL-10 in vivo treatment of MΦ-depleted mdx^ITGAM-DTR^ mice. (**A**) Representative images of Hematoxilin/Eosin staining on cryosections of TA muscle derived from mdx^ITGAM-DTR^ mice injected with DT or DT+IL-10 as described in Fig 5K. Scale bar = 200 μm. (**B-C**) Representative images of Sirius red staining on TA cryosections derived from mdx^ITGAM-DTR^ mice injected with DT or DT+IL-10 as described in Fig 5K. In the graph is reported the percentage of fibrotic area; values are mean ± SEM; n = 3 animals for each group; unpaired t test was used for comparison (P = 0.063). Scale bar = 200 μm. (**D**) In the graph is reported the percentage of Oil Red O positive area of mdx^ITGAM-DTR^ mice injected with DT or DT+IL-10; values are mean ± SEM; n = 3 animals for each group; unpaired t test was used for comparison (n.s. = not significant). (**E**) Relative percentage of SCs sorted from muscles of mdx^ITGAM-DTR^ mice injected with DT or DT+IL-10 as described in Fig 5K. SCs were sorted as α7Integrin^+^/Sca1^-^/ Lin^-^; the percentage of cells is reported as relative to whole mononucleated cells; values are mean ± SEM (n = 3 biological replicates for each experimental group). (**F**) Representative images of *in vitro* culture of α7Sca1 cells isolated from mdx^ITGAM-DTR^ mice injected with DT or DT+IL-10. α7Sca1 cells were sorted as double positive cell population α7^+^/ Sca1^+^/Lin^-^. The cells were cultured in SCs growth medium for 36 hours and then cells were fixed and stained by Oil Red O dye and counterstained with Hematoxilin.
(TIF)

S1 Table List of primers. Primers for genotyping by standard PCR or TaqMan-based qPCR; Murine expression primers for qRT-PCR analysis.
(DOCX)

S2 Table List of antibodies. FACS: Fluorescence-Activated Cell Sorting; IF: Immunofluorescence; CC: Cell Culture.
(DOCX)

S1 Data Numerical data. Numerical data underlying graphs and statistics.
(XLS)
