# Supplementary figures and images for: Macrophages fine tune satellite cell fate in dystrophic skeletal muscle of mdx mice
Source: PLoS Genet. 2019 Oct 18;15(10):e1008408. doi: 10.1371/journal.pgen.1008408 (PMC6821135; doi:10.1371/journal.pgen.1008408)

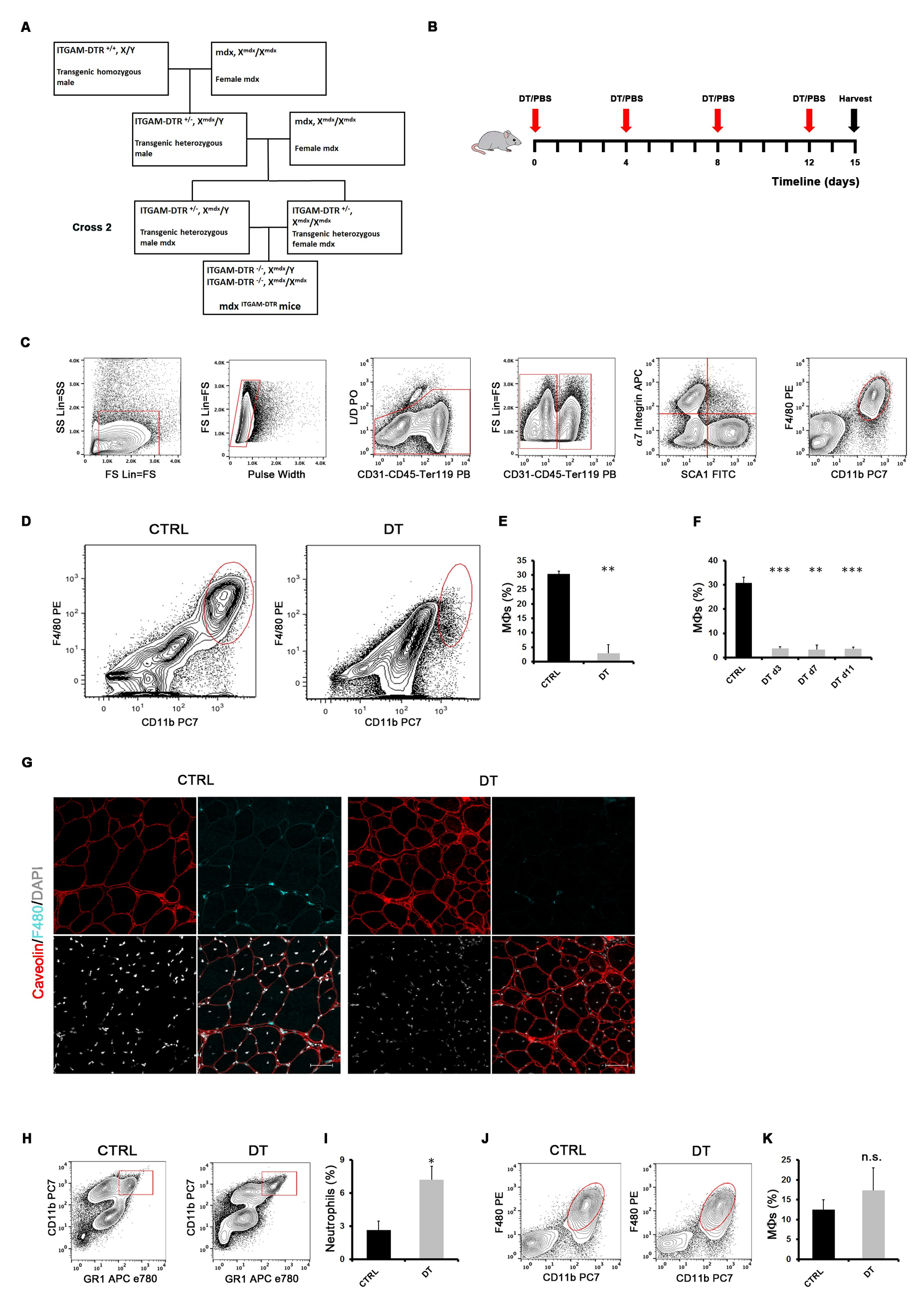

Supplement: S1 Fig — (A) Breeding scheme followed for the generation of mdxITGAM-DTR mice, by crossing mdx mice and ITGAM-DTR mice. (B) Experimental scheme for 15-day MΦ depletion in mdxITGAM-DTR mice. Either DT or PBS as vehicle, was administered by intramuscular (im) injection every 4 days in 10 week-old mice. DT was injected at 1 ng/g body weight, one injection in Tibialis Anterior (TA) muscles and two injections in Gastrocnemius (GA) muscles, 4 injections total; the mice were sacrificed 15 days (d15) after the first injection. (C) Flow cytometry gating strategy used to purify SCs, FAPs and MΦs from hind limb GA muscles of mdxITGAM-DTR mice. FSC x SSC gating was used to obtain mononuclear cells on the basis of size and granularity. Live/dead (LD) Aqua marker was used to identify live cells (Aqua negative cells). Staining with anti-hematopoietic lineage (Lin) antibodies, anti- CD31, CD45 and Ter-119 was performed to separate Lin+ from Lin-. From Lin- subpopulation, SCs was purified as α7integrin+ (APC), which are negative for Sca1 (FITC). FAPs was identified as Sca1+ (FITC) α7integrin- cells. From Lin+ subpopulation, macrophages, which are CD31-, CD45+ and Ter-119- was identified as CD11b+ (PC7) and F4/80+ (PE) double positive cells. (D-E) FACS plot showing MΦ population in mdxITGAM-DTR mice im injected with PBS (CTRL) or DT. The mice were sacrificed 15 days after the first intramuscular (im) injection of DT (1 ng/g body weight), one injection in TA muscles and two injections in GA muscles; the DT injection has been repeated every 4 days (see Experimental scheme in S1B Fig. MΦs were sorted from TA and GA muscles as Lin+/CD11b+/F4/80+ cells; in the graph is reported the percentage of MΦs expressed as relative to whole mononucleated cells; values are mean ± SEM; n = 6 animals for each group; unpaired t test was used for comparison (**, P<0.01;). (F) Graph showing MΦ depletion in mdxITGAM-DTR mice at d3, d7, d11 along the schedule of DT injection reported in S1B Fig. MΦs were sorted from [file pgen.1008408.s001.tif]

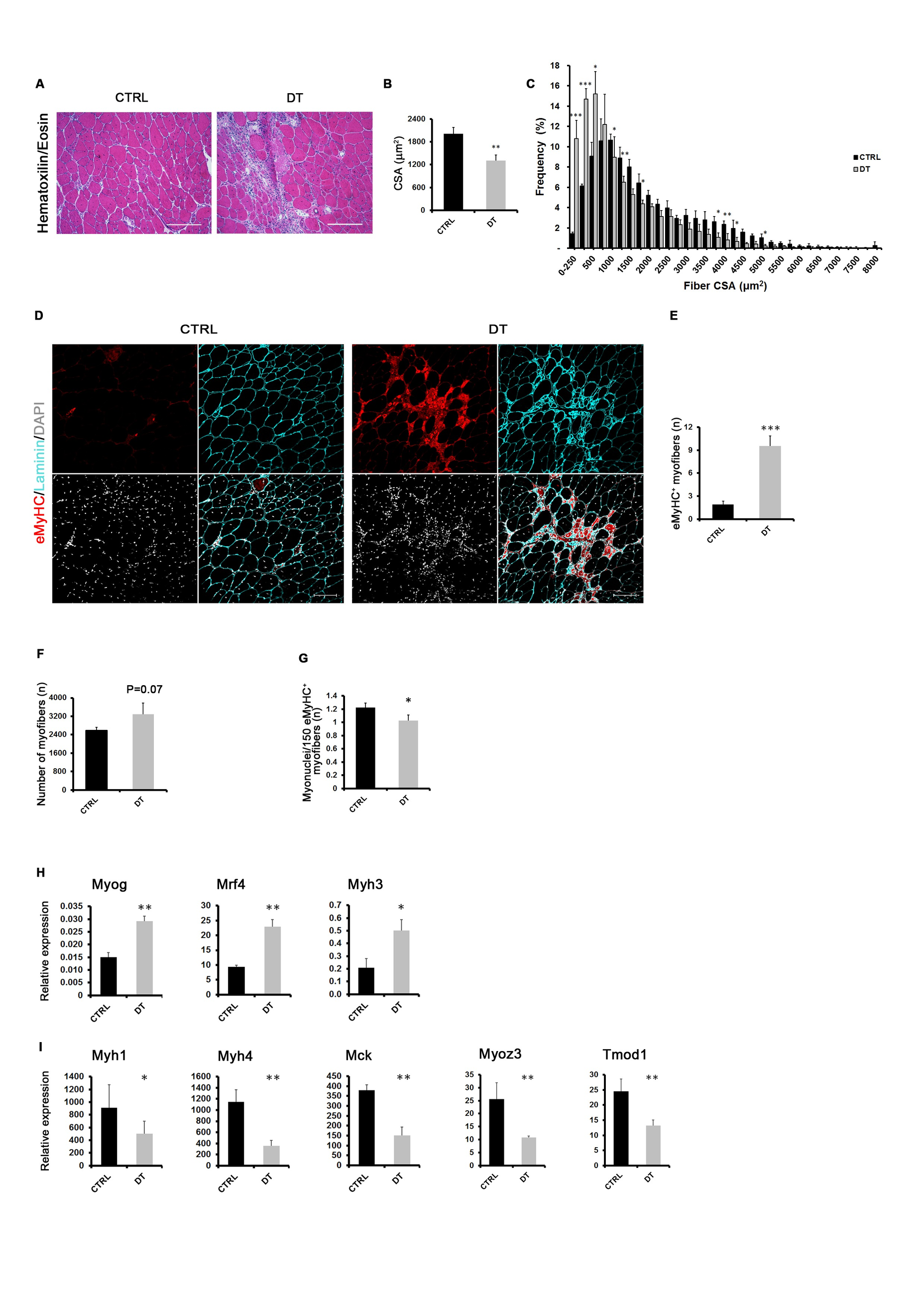

Supplement: S2 Fig — (A) Representative images of Hematoxilin/Eosin staining on cryosections of TA muscle derived from mdxITGAM-DTR mice injected with PBS or DT. Scale bar = 200 μm. (B) Mean Cross Sectional Area (CSA) of muscle fibers, measured on laminin-stained cryosections. Values are mean ± SEM (n = 3 animals for each experimental group); unpaired t test was used for comparison (**, P<0.01). (C) Frequency distribution of muscle fibers CSA of mdxITGAM-DTR mice injected with PBS or DT. Values are mean ± SEM (n = 3 animals for each experimental group); unpaired t test was used for comparison (*, P<0.05; **, P<0.01; ***, P<0.001). (D, E) Representative images of double staining anti-laminin (cyan) and anti-eMyHC (red) of TA cryosections of mdxITGAM-DTR mice injected with PBS or DT. Nuclei were counterstained with DAPI (white); n = 3 animals for each experimental group. Scale bar = 100 μm. In the graph is reported the percentage of eMyHC positive myofibers relative to total cells; values are mean ± SEM; n = 3 animals for each experimental group; unpaired t test was used for comparison (***, P<0.001). (F, G) In the graphs are reported the total number of myofibers per section (p = 0.07) (F) and the number of myonuclei/150 eMyHC+ myofibers, measured on laminin-eMyHC co-stained cryosections. (G) Values are mean ± SEM (n = 3 animals for each experimental group); unpaired t test was used for comparison (*, P<0.05). (H, I) Expression analysis of muscle markers by qRT-PCR on whole muscle (TA) derived from mdxITGAM-DTR mice injected with PBS or DT. Expression data are reported as relative to housekeeping gene TBP, and represented as mean ±SEM (n = 3–7 biological replicates for each experimental group); unpaired t test was used for comparison (*, P<0.05; **, P<0.01). (TIF) [file pgen.1008408.s002.tif]

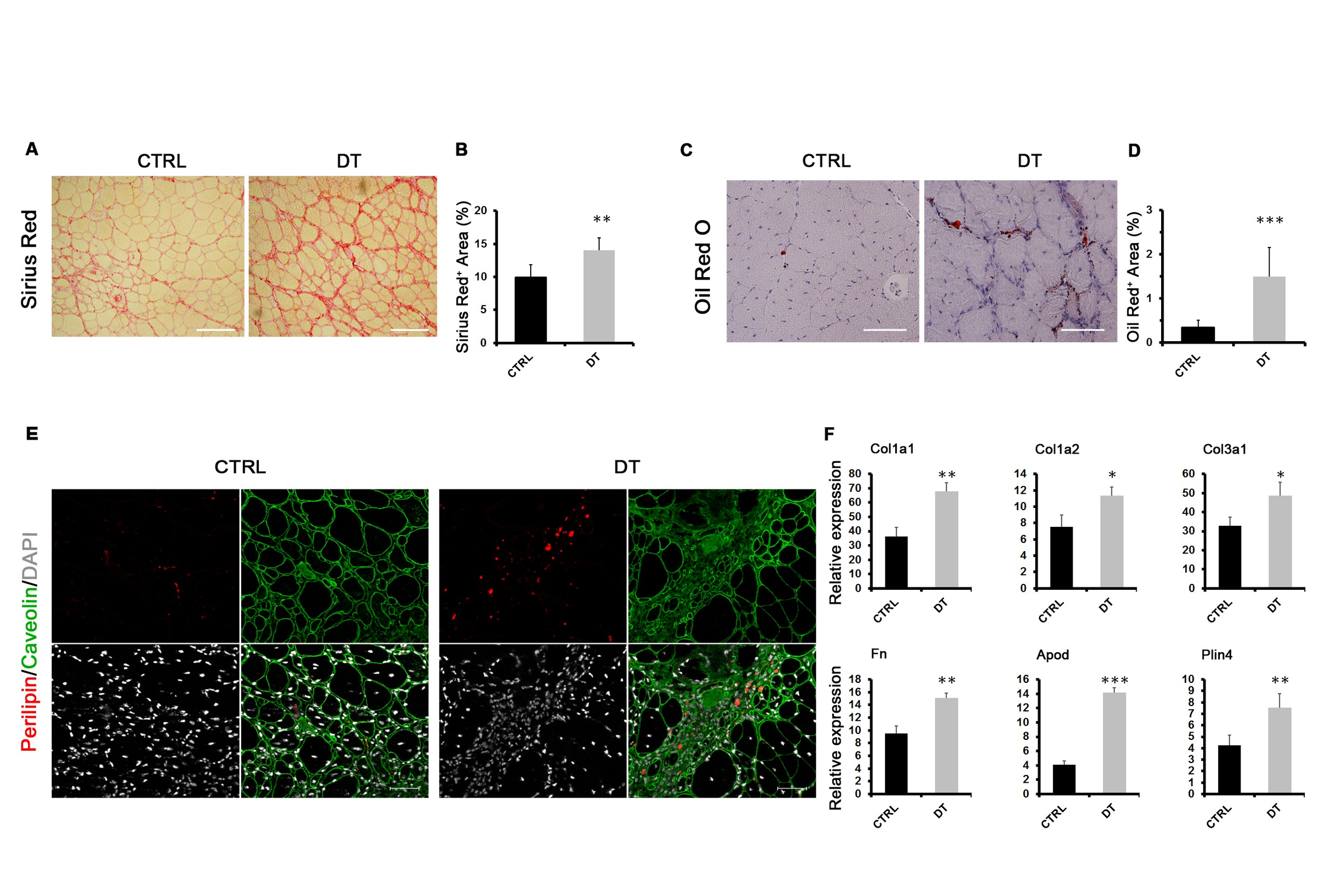

Supplement: S3 Fig — (A-B) Representative images of Sirius red staining on TA cryosections derived from mdxITGAM-DTR mice injected with vehicle (CTRL) or DT as described in S1B Fig. In the graph is reported the percentage of fibrotic area; values are mean ± SEM; n = 7 animals for each group; unpaired t test was used for comparison (**, P<0.01). Scale bar = 200 μm. (C, D) Representative images of Oil Red O staining on TA cryosections of mdxITGAM-DTR mice injected with PBS (CTRL) or DT; n = 7 animals for each group. In the graph is reported the percentage of Oil Red O positive area; values are mean ± SEM; n = 7 animals for each group; unpaired t test was used for comparison (***, P<0.001). Scale bar = 100 μm. (E) Representative images of double staining anti caveolin (green) and anti perilipin (red) of TA cryosections of mdxITGAM-DTR mice injected with PBS (CTRL) or DT. Nuclei were counterstained with DAPI (white); n = 3 animals for each group. Scale bar = 50 μm. (F) Expression analysis of fibrosis and adipogenesis markers by qRT-PCR on whole muscle (TA) derived from mdxITGAM-DTR mice injected with PBS (CTRL) or DT. Expression data are reported as relative to housekeeping gene TBP, and represented as mean ± SEM (n = 6 biological replicates for each experimental group); unpaired t test was used for comparison (*, P<0.05; **, P<0.01; ***, P<0.001). (TIF) [file pgen.1008408.s003.tif]

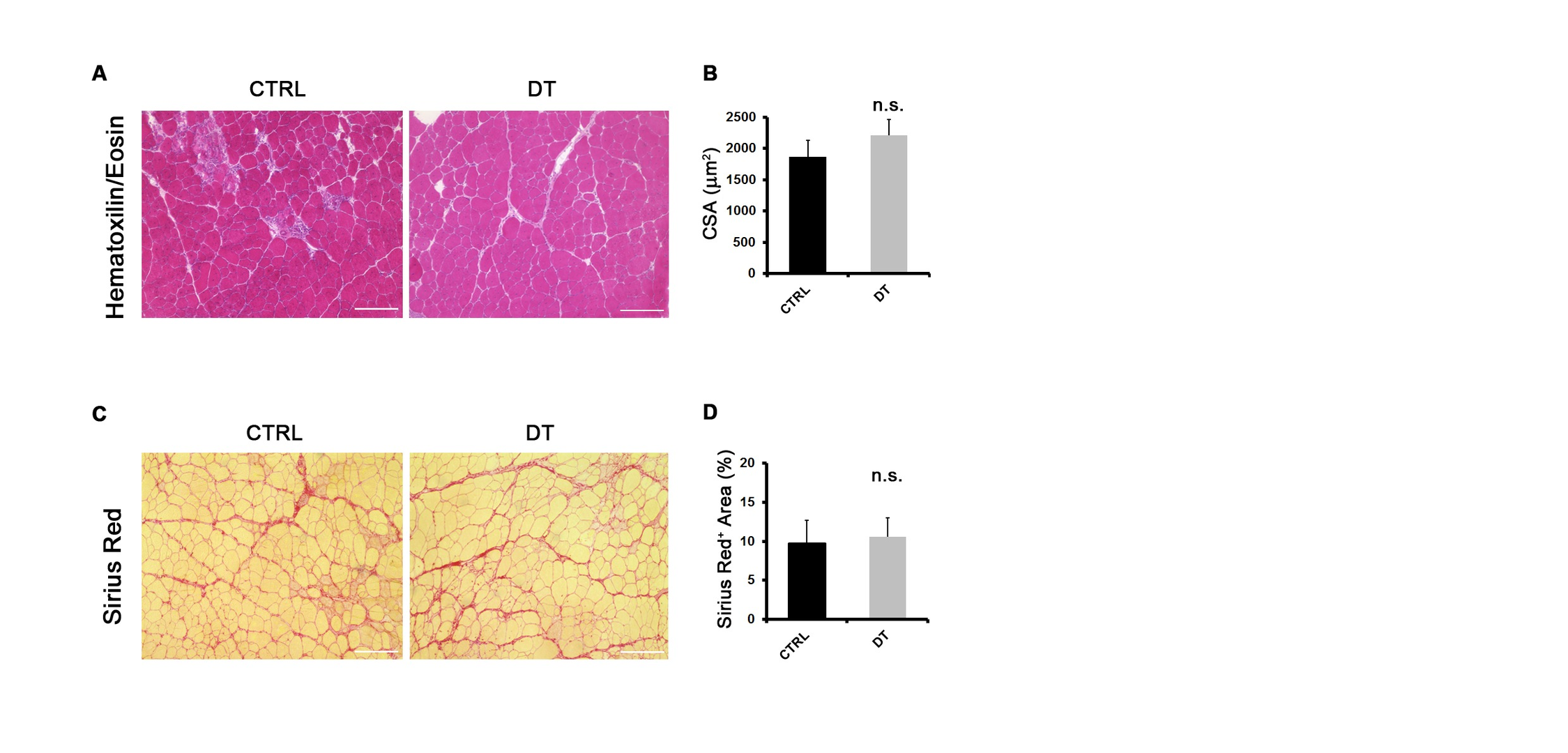

Supplement: S4 Fig — (A, B) Representative images of Hematoxilin/Eosin staining on TA cryosections derived from mdx mice injected with PBS or DT. In the graph is reported the mean of the Cross Sectional Area (CSA) of muscle fibers, measured on laminin-stained TA cryosections of mdx mice. Values are mean ± SEM (n = 3 animals for each experimental group); unpaired t test was used for comparison (n.s. = not significant). Scale bar = 200 μm. (C, D) Representative images of Sirius red staining on TA cryosections derived from mdx mice injected with vehicle (CTRL) or DT as described in S1B Fig. In the graph is reported the percentage of Sirius Red positive area; values are mean ± SEM (n = 4 PBS and 5 DT animals for each experimental group); unpaired t test was used for comparison (n.s. = not significant). Scale bar = 200 μm. (TIF) [file pgen.1008408.s004.tif]

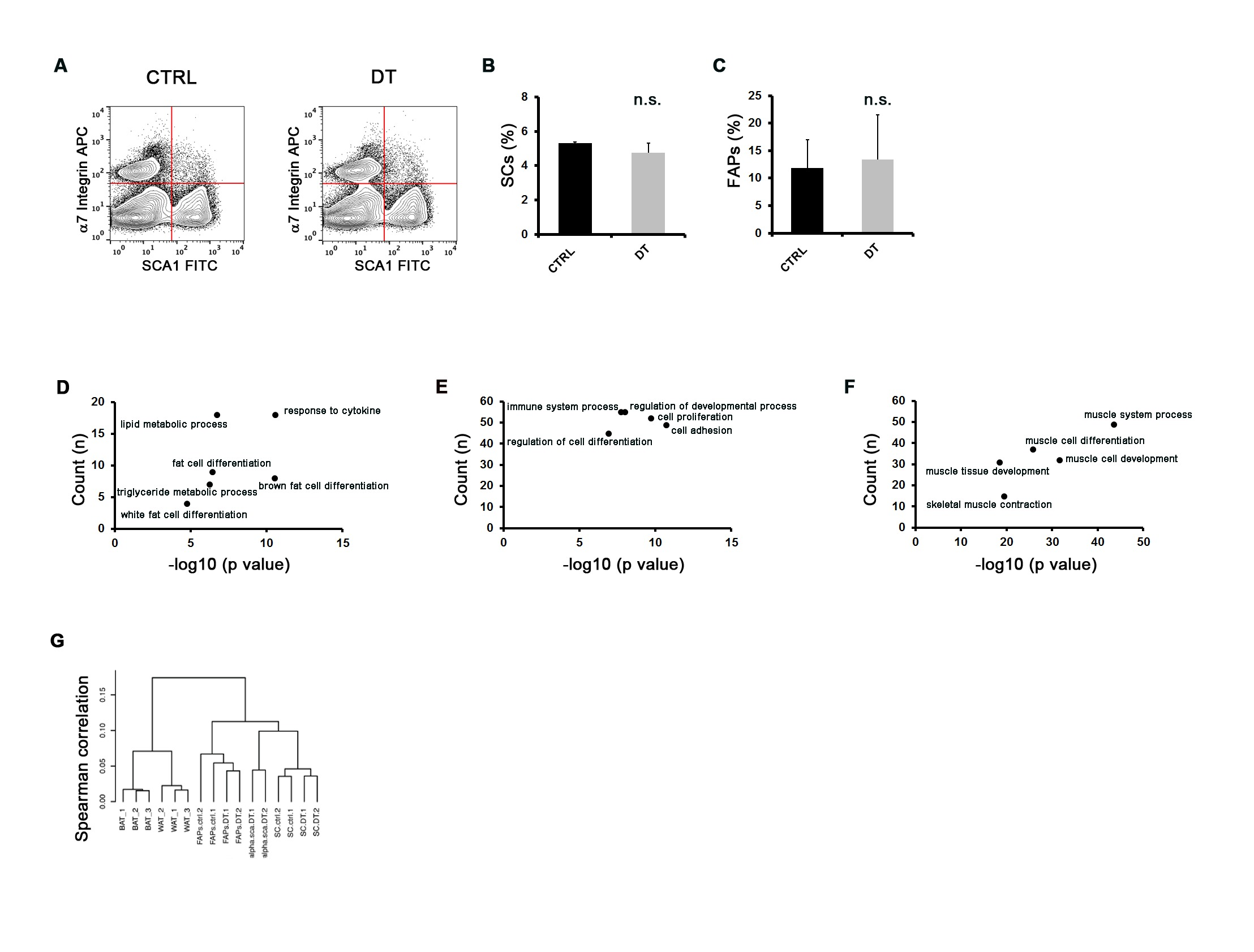

Supplement: S5 Fig — (A, B, C) FACS plot showing SCs, FAPs and α7Sca1 cell populations isolated from mdx mice im injected with PBS (CTRL) or DT (DT). Cells isolated from GA muscles were first separated into hematopoietic lineage positive (Lin+) and hematopoietic lineage negative (Lin-) (Lin: CD45, CD31 and Ter119) cells. SCs and FAPs were sorted as Lin-/α7int+/Sca1- and Lin-/Sca1+/α7int-/ cells, respectively. α7Sca1 cells (α7+/ Sca1+/Lin-) was not detectable in mdx mice. In the graphs are reported the percentage of SCs and FAPs expressed as relative to whole mononucleated cells; values are mean ± SEM; n = 4 animals for each group; unpaired t test was used for comparison (n.s. = not significant). (D, E, F) Selected representative GO biological processes in Cluster I (C), Cluster II (D) and Cluster III, as indicated in the heat map in Fig 5K and identified by DAVID 6.8. The graph displays for each GO term the obtained p value (expressed as −log10) on the x axis and the number of genes included (count), on the y axis. (G) Hierarchical clustering comparing the expression patterns of SCs, FAPs, α7Sca1 cells, BAT (brown adipose tissue) and WAT (white adipose tissue). BAT and WAT RNA-Seq samples (in triplicate) were downloaded from the GSE56367 GEO series; raw reads were filtered and aligned to mm9 similarly to the samples generated by us (SCs, FAPs, α7Sca1 cells), and absolute RPKMs for 21K genes were determined. Absolute RPKMs were normalized altogether. (TIF) [file pgen.1008408.s005.tif]

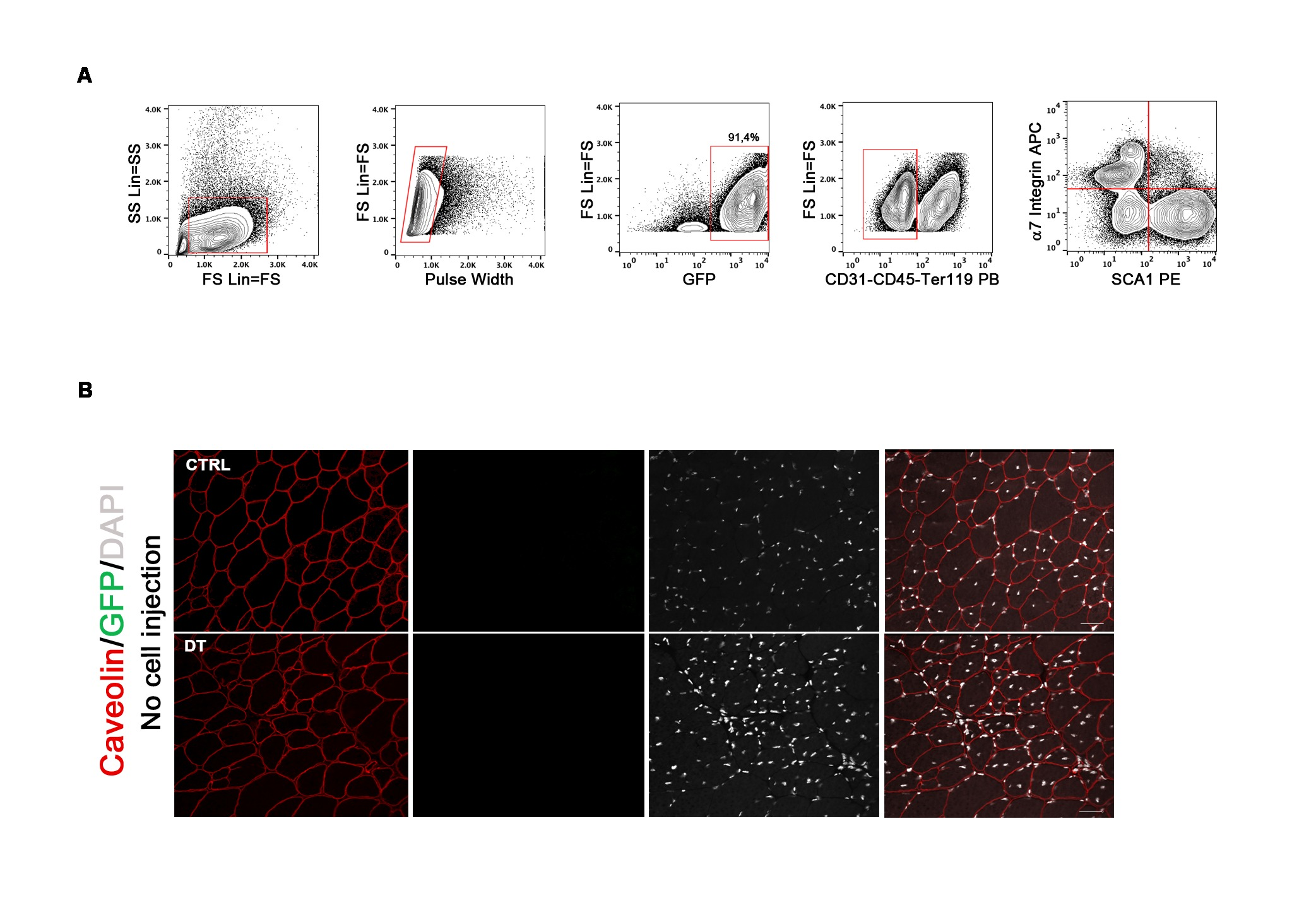

Supplement: S6 Fig — (A) Flow cytometry gating strategy used to purify SCsGFP and FAPsGFP from hind limb GA muscles of mdxGFP mice. FSC x SSC gating was used to obtain mononuclear cells on the basis of size and granularity. GFP+ cells were stained with and with lineage-specific (Lin) antibodies CD31, CD45 and Ter-119 to separate Lin+ from Lin-. From GFP+/Lin- subpopulation, SCs were purified as α7integrin+ (APC), which are negative for Sca1 (FITC). FAPs were identified as Sca1+ (FITC) α7integrin- cells. (B) Representative images of double staining anti-caveolin (red) and anti-GFP (green) of TA cryosections of mdxITGAM-DTR mice no-cell transplanted and injected with PBS (CTRL) or DT. Nuclei were counterstained with DAPI (white); n = 3 animals for each experimental group. Scale bar = 50 μm. (TIF) [file pgen.1008408.s006.tif]

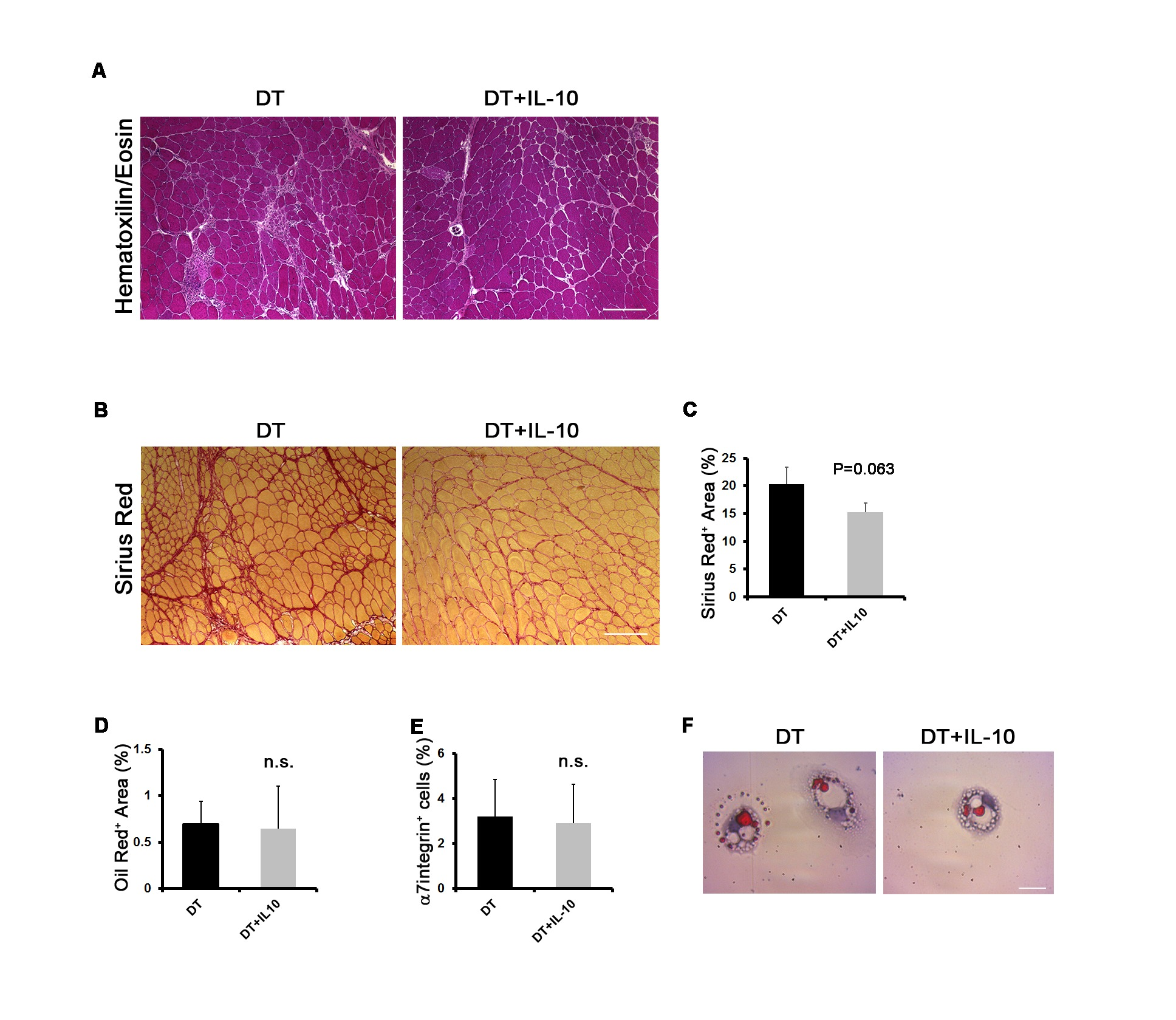

Supplement: S7 Fig — (A) Representative images of Hematoxilin/Eosin staining on cryosections of TA muscle derived from mdxITGAM-DTR mice injected with DT or DT+IL-10 as described in Fig 5K. Scale bar = 200 μm. (B-C) Representative images of Sirius red staining on TA cryosections derived from mdxITGAM-DTR mice injected with DT or DT+IL-10 as described in Fig 5K. In the graph is reported the percentage of fibrotic area; values are mean ± SEM; n = 3 animals for each group; unpaired t test was used for comparison (P = 0.063). Scale bar = 200 μm. (D) In the graph is reported the percentage of Oil Red O positive area of mdxITGAM-DTR mice injected with DT or DT+IL-10; values are mean ± SEM; n = 3 animals for each group; unpaired t test was used for comparison (n.s. = not significant). (E) Relative percentage of SCs sorted from muscles of mdxITGAM-DTR mice injected with DT or DT+IL-10 as described in Fig 5K. SCs were sorted as α7Integrin+/Sca1-/ Lin-; the percentage of cells is reported as relative to whole mononucleated cells; values are mean ± SEM (n = 3 biological replicates for each experimental group). (F) Representative images of in vitro culture of α7Sca1 cells isolated from mdxITGAM-DTR mice injected with DT or DT+IL-10. α7Sca1 cells were sorted as double positive cell population α7+/ Sca1+/Lin-. The cells were cultured in SCs growth medium for 36 hours and then cells were fixed and stained by Oil Red O dye and counterstained with Hematoxilin. (TIF) [file pgen.1008408.s007.tif]
