# Supplementary material for: Macrophages fine tune satellite cell fate in dystrophic skeletal muscle of mdx mice
Source: PLoS Genet. 2019 Oct 18;15(10):e1008408. doi: 10.1371/journal.pgen.1008408 (PMC6821135; doi:10.1371/journal.pgen.1008408)
Supplement: S1 Table — Primers for genotyping by standard PCR or TaqMan-based qPCR; Murine expression primers for qRT-PCR analysis. (DOCX) [file pgen.1008408.s008.docx]

**S1 Table. List of Primers**

| **Primers for genotyping** | | | |
| --- | --- | --- | --- |
| **Primer Name** | **Forward (Sequence 5’- 3’)** | **Reverse (Sequence 5’- 3’) (Sequence 5’- 3’) (Sequence 5’- 3’)** | **Application** |
| ITGAM Transgene | GGGACCATGAAGCTGCTGCCG | TCAGTGGGAATTAGTCATGCC | PCR |
| Internal Positive Control | CAAATGTTGCTTGTCTGGTG | GTCAGTCGAGTGCACAGTTT | PCR |
| ITGAM Transgene (qPCR) | AGTGCTTCAGCCGCTACC | GAAGATGGTGCGCTCCTG | qPCR |
| ITGAM Transgene Probe (FAM) | TTCAAGTCCGCCATGCCCGAA |  | qPCR |
| Internal Positive Control (qPCR) | CACGTGGGCTCCAGCATT | TCACCAGTCATTTCTGCCTTTG | qPCR |
| Internal Control Probe (Cy5) | CCAATGGTCGGGCACTGCTCAA |  | qPCR |
| **Primers for RT-qPCR** |  |  |  |
| **Gene symbol** | **Forward (Sequence 5’- 3’)** | **Reverse (Sequence 5’- 3’)** | **Application** |
| Myh3 | CAATAAACTGCGGGCAAAGAC | CTTGCTCACTCCTCGCTTTCA | qRT-PCR |
| Myog | GTCCCAACCCAGGAGATCATTT | CAGACATATCCTCCACCGTGA | qRT-PCR |
| Mrf4 | ACCCCTACAGCTACAAACCC | ACGTTTGCTCCTCCTTCCTT | qRT-PCR |
| Myh1 | GTTCCTCCTTCCAGACCGTG | GGGGATGATGCACCGTACAA | qRT-PCR |
| Myh4 | GTCCTTCCTCAAACCCTTAAAGT | CATCTCAGCGTCGGAACTCA | qRT-PCR |
| Mck | CTTCCTGTTTGACAAGCCCG | CTCCTCGTTCACCCACACAA | qRT-PCR |
| Tmod1 | AGGGGAGAAACGAGGAAAGG | CACACAGTTCTGCATCCGAG | qRT-PCR |
| Myoz3 | CAGGAGTTCACCAGCTACCA | GGGTCTTGTTGAAGTTGCGA | qRT-PCR |
| Col1a1 | CCTCAGGGTATTGCTGGACA | GAAGGACCTTGTTTGCCAGG | qRT-PCR |
| Col1a2 | GGAACAAATGGGCTCACTGG | CAAGTCCTCTGGCACCTGTA | qRT-PCR |
| Col3a1 | CCCAACCCAGAGATCCCATT | GGTCACCATTTCTCCCAGGA | qRT-PCR |
| Fn | TCCACGCCATTCCTGCGCC | GCACCCGGTAGCCAGTGAG | qRT-PCR |
| Apod | AAGCTCGCTGGGATCTTCTC | AATTTCCATCTTGGGAAATGC | qRT-PCR |
| Plin4 | ACCAACTCACAGATGGCAGG | AGGCATCTTCACTGCTGGTC | qRT-PCR |
| C/Ebpα | GTCACTGGTCAACTCCAGCA | TGGACAAGAACAGCAACGAG | qRT-PCR |
| Pparγ | CGCTGATGCACTGCCTATGA | AGAGGTCCACAGAGCTGATTCC | qRT-PCR |
| Pax7 | GCCGAGTGCTCAGAATCAA | AGCCCTCATCCAGACGGTT | qRT-PCR |
| Myf5 | TCAAATGCATGTGCTGCAGATAA | GCTCGGATGGCTCTGTAGAC | qRT-PCR |
| Myod1 | GTCGTAGCCATTCTGCCG | AGCACTACAGTGGCGACTCA | qRT-PCR |
| Myh1 | GTTCCTCCTTCCAGACCGTG | GGGGATGATGCACCGTACAA | qRT-PCR |
| Myh4 | GTCCTTCCTCAAACCCTTAAAGT | CATCTCAGCGTCGGAACTCA | qRT-PCR |
| Mck | CTTCCTGTTTGACAAGCCCG | CTCCTCGTTCACCCACACAA | qRT-PCR |
| Ccnd1 | TCCTCTCCAAAATGCCAGAG | GGGTGGGTTGGAAATGAAC | qRT-PCR |
| Ccna2 | AGAGTGTGAAGATGCCCTGG | GTGGTGATTCAAAACTGCCA | qRT-PCR |
| Cdk4 | TGCCAGAGATGGAGGAGTCT | TTGTGCAGGTAGGAGTGCTG | qRT-PCR |
| Numb | CCAGTTAAGTACCTCGGCCA | CGGCCTTCACTGCTTTCTTT | qRT-PCR |
| Lix | GCTAAAGTGGCGCTCATCAA | CACTTGTGCTTGGGTCATCC | qRT-PCR |
| Spry | GTCTTCTGAGCAGGGCCTAT | AGGAGGCCTTCAAGTCTTCC | qRT-PCR |
| AdipoQ | GGTCCTAAGGGTGAGACAGG | GAGCGATACACATAAGCGGC | qRT-PCR |
| Fabp4 | CAGCCTTTCTCACCTGGAAG | GGTGACCAAATCCCCATTTA | qRT-PCR |
| Ucp1 | GGAGAGAAACACCTGCCTCT | CTGACCTTCACGACCTCTGT | qRT-PCR |
| Cidea | TCTGCAATCCCATGAATGTC | CAGTGATTTAAGAGACGCGG | qRT-PCR |
| Tbp | CTGGAATTGTACCGCAGCTT | TCCTGTGCACACCATTTTTC | qRT-PCR |
