# Supplementary material for: Macrophages fine tune satellite cell fate in dystrophic skeletal muscle of mdx mice
Source: PLoS Genet. 2019 Oct 18;15(10):e1008408. doi: 10.1371/journal.pgen.1008408 (PMC6821135; doi:10.1371/journal.pgen.1008408)
Supplement: S2 Table — FACS: Fluorescence-Activated Cell Sorting; IF: Immunofluorescence; CC: Cell Culture. (DOCX) [file pgen.1008408.s009.docx]

**S2 Table. List of Antibodies**

| **Antibody** | **Company, Cat #** | **Dilution** | **Application** |
| --- | --- | --- | --- |
| CD31-Pacific blue, clone # 390 | Life Technologies, RM5228 | 1:50 | FACS |
| CD45-Pacific blue, clone #30-F11 | eBioscience, Cat#: MCD4528 | 1:50 | FACS |
| Ter119-Pacific blue, clone TER-119 | eBioscience, Cat#: 48-5921-82 | 1:50 | FACS |
| CD11b-PECy7, clone M1/70 | BD Biosciences, cat#552850 | 1:200 | FACS |
| F480-PE, clone BM8 | ThermoFisher Cat#: 14-4801-81 | 1:50 | FACS |
| α7-integrin-APC-647, clone R2F2 | AbLab Cat#: AB10RS24MW215 | 1:500 | FACS |
| Sca1-FITC, clone D7 | eBioscience, Cat#: 11-5981-81 | 1:50 | FACS |
| Gr1-APC-e780 | eBioscience, BMS47-5931-80 | 1:200 | FACS |
| SCA1-PE, clone D7 | eBioscience Cat#. 12-5981-83 | 1:50 | FACS |
| eMyHC, clone F1.652 | DSHB: Developmental Studies Hybridoma Bank | 1:20 | IF |
| Laminin | Sigma-Aldrich Cat#: L9393 | 1:400 | IF |
| Perilipin | Sigma-Aldrich Cat#: P1873 | 1:200 | IF |
| Caveolin-3 | BD Transduction Laboratories Cat#: 610420 | 1:400 | IF |
| Pax7 | DSHB: Developmental Studies Hybridoma Bank | 1:20 | IF |
| F480 | Bio-Rad Cat#: MCA497G | 1:150 | IF |
| Col1 | AbCam Cat#: ab6308 | 1:200 | IF |
| GFP | AbCam Cat#: ab6556 | 1:400 | IF |
| GFP | Santa Cruz Cat#. 9996 | 1:200 | IF |
| MyHC, MF20 | DSHB: Developmental Studies Hybridoma Bank | 1:20 | IF |
| Myog F5D | DSHB: Developmental Studies Hybridoma Bank | 1:20 | IF |
| Laminin | Alexis, ALX-804-190-C100 | 1:500 | IF |
| IL-10 | BioLegend Cat#. 504904 | 2.5 μg/ml | CC |
| IgG | BioLegend Cat#. 400427 | 2.5 μg/ml | CC |
